# Evaluation of the effect of Cooled HaEmodialysis on Cognitive function in patients suffering with end-stage KidnEy Disease (E-CHECKED): feasibility randomised control trial protocol

**DOI:** 10.1186/s13063-020-04725-0

**Published:** 2020-09-30

**Authors:** Indranil Dasgupta, Aghogho Odudu, Jyoti Baharani, Niall Fergusson, Helen Griffiths, John Harrison, Paul Maruff, G Neil Thomas, Gavin Woodhall, Samir Youseff, George Tadros

**Affiliations:** 1 Renal Unit, Heartlands Hospital, Bordesley Green East, Birmingham, B9 5SS UK; 2 Warwick Medical School, University of Warwick, Coventry, UK; 3 Division of Cardiovascular Sciences, University of Manchester, Manchester, UK; 4 Manchester University NHS Foundation Trust, Manchester, UK; 5 Department of Care of the Elderly, Heartlands Hospital, Birmingham, UK; 6 Faculty of Health and Medical Sciences, University of Surrey, Guildford, UK; 7 Institute of Psychiatry, Psychology & Neuroscience, King’s College London, London, UK; 8 Cogstate Limited, Melbourne, Australia; 9 Institute of Applied Health Research, University of Birmingham, Birmingham, UK; 10 School of Neuropharmacology, Aston University, Birmingham, UK; 11 Patient Representative, Birmingham, UK; 12 Department of Old Age Psychiatry, Heartlands Hospital, Birmingham, UK; 13 Aston Medical School, Aston University, Birmingham, UK

**Keywords:** Cognition, Cognitive function, Cold temperature, Haemodialysis, Haemodialysis solutions, Randomised controlled trial

## Abstract

**Background:**

Cognitive impairment is common in haemodialysis (HD) patients and is associated independently with depression and mortality. This association is poorly understood, and no intervention is proven to slow cognitive decline. There is evidence that cooler dialysis fluid (dialysate) may slow white matter changes in the brain, but no study has investigated the effect of cooler dialysate on cognition. This study addresses whether cooler dialysate can prevent the decline in cognition and improve quality of life (QOL) in HD patients.

**Methods:**

This is a multi-site prospective randomised, double-blinded feasibility trial. Setting: Four HD units in the UK. Participants and interventions: Ninety HD patients randomised (1:1) to standard care (dialysate temperature 36.5 °C) or intervention (dialysate temperature 35 °C) for 12 months. Primary outcome measure: Change in cognition using the Montreal Cognitive Assessment (MoCA). Secondary outcome measures: Recruitment and attrition rates, reasons for non-recruitment, frequency of intradialytic hypotension, depressive symptom scores, patient and carers burden, a detailed computerised cognitive test and QOL assessments. Analysis: mixed method approach, utilising measurement of cognition, questionnaires, physiological measurements and semi-structured interviews.

**Discussion:**

The results of this feasibility trial will inform the design of a future adequately powered substantive trial investigating the effect of dialysate cooling on prevention and/or slowing in cognitive decline in patients undergoing haemodialysis using a computerised battery of neuro-cognitive tests. The main hypothesis that would be tested in this future trial is that patients treated with regular conventional haemodialysis will have a lesser decline in cognitive function and a better quality of life over 1 year by using cooler dialysis fluid at 35 °C, versus a standard dialysis fluid temperature of 36.5 °C. This also should reflect in improvements in their abilities for activities of daily living and therefore reduce carers’ burden. If successful, the treatment could be universally applied at no extra cost.

**Trial registration:**

ClinicalTrials.gov NCT03645733. Registered retrospectively on 24 August 2018.

## Background

Increasing severity of chronic kidney disease (CKD) is associated with a gradual increase in prevalence of cognitive impairment [1, 2] independent of vascular risk factors [3]. Different diagnostic methods influence the definition and incidence of cognitive impairment, but recent reviews suggest the presence of at least moderate cognitive impairment in 30–70% of haemodialysis (HD) patients [4–6]. Cognitive impairment in HD patients is associated independently with higher rates of depression and mortality [4, 7], although this association is likely complex and poorly understood. Severe depression can even mimic cognitive impairment making it important to measure depression rates in studies of cognitive impairment to understand the interaction. Traditional atherosclerotic risk factors [7] cannot entirely account for the excess risk of cognitive impairment [8]. Multiple HD specific factors including oxidative stress, malnutrition and inflammation are implicated [9]. The process of HD does not remove toxins as efficiently as the native kidney and accumulation of several neurotoxins [10] may also act to reduce brain perfusion and compromise blood-brain barrier integrity [11]. The process of HD involves cycles of removing varying volumes of fluid, electrolytes and toxins that accumulate between treatments. Intradialytic hypotension is common affecting 30–40% of treatments and is associated with at least a 30% increase in mortality and reduced quality-of-life (QOL) [12]. Left ventricular hypertrophy and aortic stiffness are seen in most HD patients and further lower diastolic coronary perfusion. The result is that HD might be seen as a process that frequently involves repeated ischemic insults to the brain and other organs [13–15]. These dynamic changes in blood pressure (BP) and perfusion might be associated with altered cognition, but supporting data are sparse and conflicting, possibly reflecting differences in study design such as different methods and timings of cognitive assessment. Several small studies show cognition is best immediately before HD, becomes worse during HD and then improves the day after with a possible link to acute fluid removal [16, 17]. Absence of intradialytic hypotension is emerging as a novel treatment goal and it is plausible that preventing intradialytic hypotension might prevent intradialytic brain ischemia and slow the development of cognitive impairment in HD patients [18]. One method that may prevent intradialytic hypotension is to increase treatment time or frequency to allow a slower rate of fluid removal [19]. A preliminary repeated measures study of 12 patients showed extended overnight HD was associated with improved cognition measured by a battery of neuropsychological tests [20]. These data are encouraging but come at the expense of increased treatment complications, cost and are currently not feasible in most centres in the UK and worldwide. The use of cooler dialysate (34–35 °C) to prevent intradialytic hypotension was first described in 1981 [21], but remains underused because of perceptions about thermal symptoms [22–24]. Cooler dialysate is thought to prevent intradialytic hypotension by preventing a rise in core temperature and subsequent systemic vasodilation [25]. A recent systematic review showed that compared with standard temperature dialysis, cooler dialysis reduced the event rate of intradialytic hypotension by 70% (95% CI, 49–89%) [24]. A recent pilot clinical trial in 38 patients showed lower temperature of dialysis fluid applied for one year prevented worsening of baseline ischaemic brain white matter change through ameliorating haemodynamic instability [26, 27]. A trial also reported cooler dialysis fluid improved cardiac structure and function [28]. While these results show the potential for dialysate cooling as a cardioprotective and neuroprotective treatment, the effects of cooler dialysate on cognition, QOL and illness burden remain unknown. There is also little information about how cooler dialysis fluid is tolerated. The current low usage of cooler dialysate in the UK affords an opportunity to definitively test this simple modification to HD as a potential intervention to prevent cognitive impairment and increase QOL. There remain several uncertainties around study design of a definitive trial of cooler dialysate and cognitive impairment; hence, there is a need to assess this formally in a feasibility study.

## Methods

We aim to perform a feasibility trial that will inform the development of a definitive, fully powered, randomised, controlled clinical trial that would examine the efficacy of cooler dialysis fluid in reducing cognitive decline in patients receiving HD for End-Stage Kidney Disease (ESKD). The main hypothesis that would be tested in this future trial is that patients treated with regular conventional HD will have a reduced decline in cognition and a better QOL over 1 year by using cooler dialysis fluid at 35 °C, versus patients treated using a standard dialysis fluid temperature of 36.5 °C.

### Primary objective

The primary objective is to test the feasibility of the investigation of lower temperatures of dialysis fluid in preventing the decline in cognitive function and improve the quality of life in HD patients.

### Secondary objectives

1. To provide an estimate of the variability in the outcome measures for the cooled dialysis and standard treatment arms, to inform a future, adequately powered, definitive trial.
2. To measure the frequency of intradialytic hypotension as an explanatory outcome.
3. To measure recruitment and attrition rates to inform the design of a larger clinical trial.
4. To record reasons for non-recruitment and study attrition to inform the design of a larger clinical trial.
5. To measure baseline levels of depression in the targeted population to inform estimates of exclusion rates for participants with depressive pseudo cognitive impairment from the future trial.
6. To assess the burden of study-related interventions and assessments on patients and carers.
7. To assess the administration, suitability and adherence of the chosen method for assessment of cognition in patients, especially in those from ethnic minorities.
8. To assess the administration and suitability of the chosen QOL scales and activities of daily living in HD participants.
9. To assess the administration and suitability of the chosen method for measuring carers’ burden in this group.

The protocol will be considered viable for a definitive RCT without modification if the following outcomes are met:

1. Recruitment rates of at least 50% of eligible patients;
2. Attrition rate of < 20% by 6 months;
3. Compliance rate of 60% to trial.

If these outcomes are not met, the protocol will be modified in light of the study’s findings.

We used the Standard Protocol Items: Recommendations for Interventional Trials (SPIRIT) checklist when writing our report [29].

### Study design

**Fig. 1:**
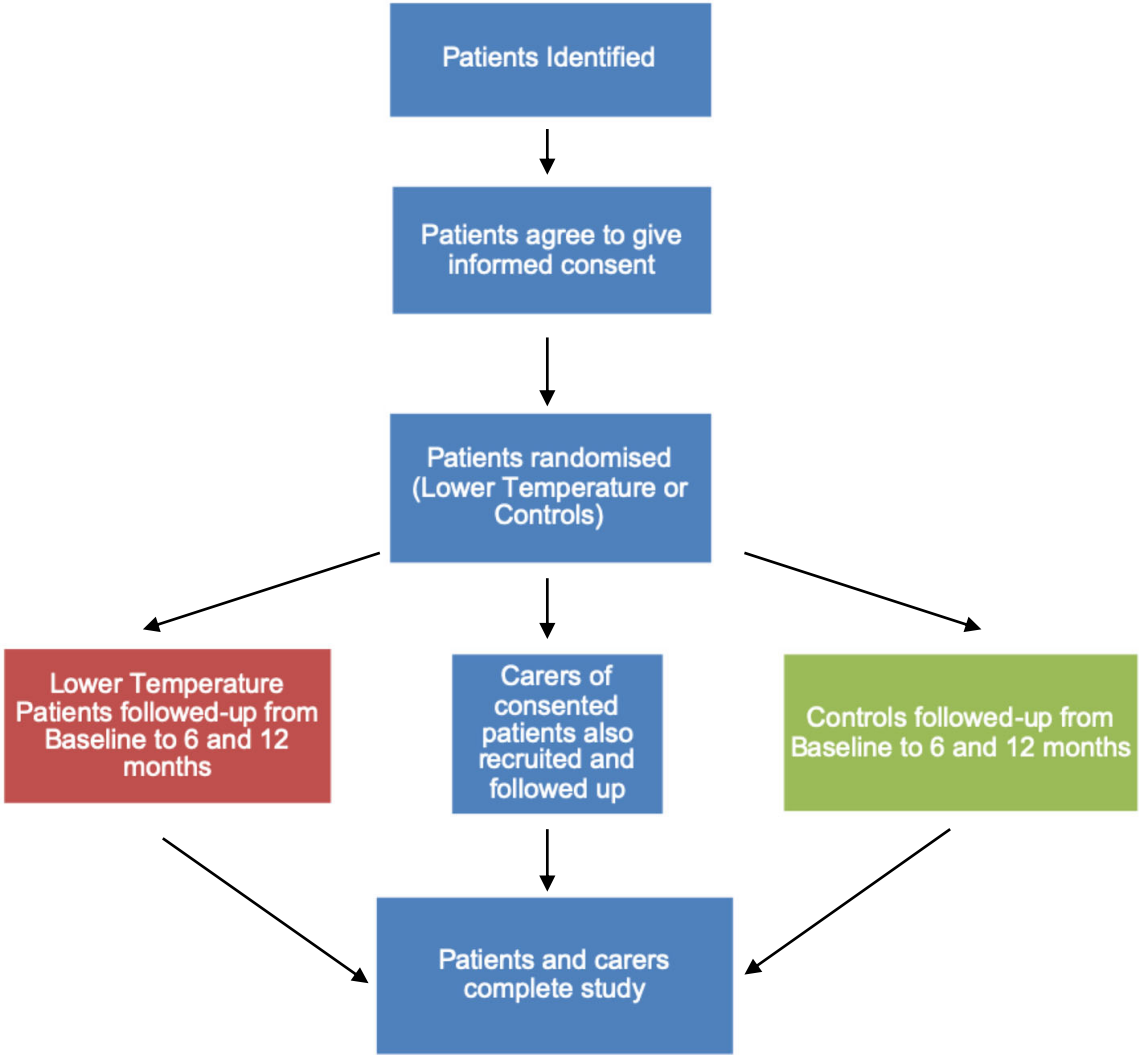
Study flow chart

The schedule of events is summarised in Fig. 1 and Table 1. The study is a multi-site, prospective, randomised, double-blinded, controlled, feasibility trial [30].

**Table 1:** Study schedule of data collection and assessments

	Baseline (O month)	6 months	12 months
Consent	X		
Randomisation	X		
Baseline data (defined)	X		
Cognitive function:			
Montreal Cognitive Assessment [31]	X	X	X
Cogstate [32]	X	X	X
Confusion Assessment Method [33]	X	X	X
Tolerability of Low Temperature Dialysis Questionnaire	Every 2 weeks for the first 6 weeks		
Activities of daily living:			
Assessment of QoL [34]	X	X	X
Bristol Activities of Daily Living Scale [35]	X	X	X
Carer burden assessment [36]	X	X	X
Hospital Anxiety and Depression Scale [37]	X	X	X
HD recovery time [38]	X	X	X
Qualitative interview			At completion or drop out
Dialysis temperature recording	During each HD session		
Physiological measurements*	During each HD session		
Laboratory measurements**	Measured monthly as part of routine care		
Adverse event reporting	X	X	X
Review Concomitant Medications	X	X	X

*MoCA* Montreal Cognitive Assessment tool, *CAM* Confusion Assessment Method, *QoL* quality of life, *ADL* activities of daily living, *HADS* Hospital Anxiety and Depression Scale

*Blood pressure (pre and post HD), intradialytic hypotension, nursing interventions for intradialytic hypotension, intradialytic weight gain over preceding 1 month

**KT/V as markers of adequate solute clearance, routine haematology and biochemistry

### Study setting and population

Patients will be recruited through the renal clinics at four HD units in the United Kingdom at Birmingham Heartlands Hospital, Runcorn Road Renal Unit, Solihull Hospital and Castle Vale Renal Unit. All these HD units are under the collective organisation of the University Hospitals Birmingham NHS Foundation Trust. The study aims to recruit 90 patients allocating 45 patients in the intervention and 45 patients in the control group. Suitable patients will be identified by the direct care team. The care team will speak with the suitable patients to see if they are happy to have their details passed on to the research team for further discussion. If the patient agrees, the research team will contact the patient and determine eligibility. Suitable patients will be identified through searching records in the four participating units. An information pack will be given to the patient (see Additional file 1), who will be given 24 h to read the information and ask any questions they may have. The research team will then contact the patient, in a pre-agreed manner (i.e. next clinic visit, via telephone) to see if they have any further questions or would like to participate in the study. If they would like to participate in the study they will arrange to meet and complete the consent form (see Additional file 2).

### Inclusion criteria

1. Aged 18 years or greater.
2. Receiving HD three times per week for ESKD, for at least 3 months.
3. Having proven mental capacity to understand the study and give informed consent.

### Exclusion criteria

1. Established diagnosis of dementia in a memory clinic or specialised service.
2. Receiving acetylcholinesterase inhibitors.
3. Receiving antipsychotic or antidepressants unless stable on treatment for at least 6 weeks.
4. Current participation in a study of an investigational medicinal product.
5. Inter-current infection.
6. An operation date for a living donor kidney transplant within the period of the trial.
7. Patients expected to survive less than 1 year according to the treating nephrologist.
8. Patients prone to intra-dialytic hypotension or cardiovascular instability during HD according to the treating nephrologist.
9. Patients who are currently taking triptans, dopamine antagonists, tramadol, sedative and opioid analgesics.
10. Patients who have a known diagnosis or have other psychiatric conditions, including severe depression, bipolar affective disorder, severe anxiety, panic disorder, substance misuse or psychosis.
11. Currently involved in another intervention study.

### Inclusion criteria (carers)

1. Adult above the age of 18.
2. Consents to take part in the study.
3. Speaks English.

### Exclusion criteria (carers)

1. Not in regular contact with the patient.
2. Any apparent personal or psychological conflicts with the patient that could skew their feedback as judged by the research team.
3. Evidence for very poor physical health that would prevent them from completing the study.

### Participant withdrawal

Participants are free to withdraw at any time and this will not affect their future care. They will be asked if they wish to withdraw completely, and have all data removed from the study, or just from the point of withdrawal.

### Randomisation and concurrent treatments

Currently in the UK, temperatures between 35 °C and 37 °C are empirically used in dialysis; however, the best temperature is not known and may be differentially tolerated depending on the patients’ own core temperature [39]. International clinical guidelines recommend a minimum temperature for the dialysate of 35 °C mainly for cardiovascular stability [40, 41]. All consenting participants eligible for inclusion will be randomised on a 1:1 basis to the control group dialysate temperature of 36.5 °C for 12 months or cooled dialysate temperature of 35 °C for 12 months. Randomisation will be stratified by age group using a secure internet-based system that concealed treatment allocations (Sealed Envelope, London, UK). The randomisation software (https://www.sealedenvelope.com/simple-randomiser/v1/trials/e-checked) contains the randomisation sequence that is not available to the research personnel (unless blinding needs to be broken in case of SAEs). The software requires the details of the patients to be entered before the randomisation arm is revealed thereby maintaining concealment of the allocation. This process will be performed by a research nurse who will not be involved with any other part of the study.

### Study interventions

Patients will be randomised to one of two groups: the intervention and the control group. Both groups will have a pre-study run-in phase of 2 weeks to establish pre-dialysis temperature with a tympanic thermometer taken at each session. The control group will then use the standard dialysate temperature of 36.5 °C. The intervention group will start off using a dialysate temperature of 36 °C. Thereafter, the dialysate temperature will be reduced every 2 weeks by 0.5 °C until a temperature of 35 °C is reached. Patients who fail to tolerate the temperature of 35 °C, the lowest tolerated temperature, will be carried over to the end of the study. Tympanic and dialysate temperature will be recorded at every session regardless of study group to aid monitoring of protocol adherence and allow an interim analysis of patient’s temperatures to ensure a clear temperature separation of the study groups. The research nurse will assess temperature tolerability every 2 weeks using a Tolerability of Low Temperature Dialysis Questionnaire for the first 6 weeks. The patients will not be informed to their group allocation nor the temperature setting of the machine to enable unbiased comparison of the tolerability of the intervention. The investigators carrying out cognitive assessment and study related procedures will also be blinded to their group allocation. The clinical nursing staff must be unblinded to deliver the intervention, but any temperature display on the machine will be concealed from the patients. Any patient from the control group and intervention group complaining of feeling cold during haemodialysis session will be provided with an extra blanket to aid tolerance and improve comfort. But if patient could not tolerate the lower temperature to the point that they felt they could terminate the session, the temperature will be increased back to the previous setting. This ensures a minimum between-group temperature separation of 0.5 °C for the expectedly few intervention group patients who can only tolerate dialysate temperature reduction to 36 °C. In a previous similarly designed study, only 2 of 73 participants required this protocol deviation [28].

### Blinding

This study design allows double blinding of both patients and investigators. All outcomes are measured at 0 (baseline), 6 and 12 months by a blinded rater, on a non-dialysis day when the best performance is expected [16, 17]. Blinding of the rater could be compromised if the rater visited the patient during HD as machine settings might be visible. Testing patients shortly before a HD treatment might be inconvenient with implications for recruitment and retention. Therefore, in this study, all assessments are conducted in domiciliary visits in the homes of patients or a mutually agreed venue. The carer assessment will also be taken at months 0, 6 and 12 by a blinded independent rater. The only person for whom blinding will not be practical is the clinical nursing staff in the haemodialysis unit setting the temperature of the machine based on the patient’s allocation. However, these staff will have no contact regarding the temperature settings with either patients or investigators performing the assessments regarding the temperature settings.

### Assessment and data collection

#### Data collection

The data collection schedule is summarised in Table 1. Once a participant has given valid informed consent, there will be three study visits at baseline, 6 and 12 months. Data collected will include baseline demographics. The research team are committed to inclusion. Local audit data showed that after English, Urdu and Bengali are the two most used native languages. Whilst inclusion criteria include a good command of English language, we will ensure where possible assessments are available in Urdu and Bengali.

#### Qualitative data and quantitative data to inform future trial design

The main aim of the qualitative component is to assess issues related to patient recruitment. This will include practicalities of implementing cooler dialysate, adherence to treatment, effectiveness of blindness process and identification of factors that may affect routine practice of treatment in various centres. We will apply thematic analysis to qualitative data collected from semi-structured interviews.

#### Tolerability of low temperature

Patients are asked about their ability to tolerate the low temperature and their level of comfort. They are also asked whether they need any extra support. The questionnaire was locally designed and used in an unpublished pilot study where it showed face validity and reliability.

#### Confusion Assessment Method (CAM)

The Confusion Assessment method (CAM) is a valid tool that is brief to administer and excludes the effect of delirium on cognitive performance [33]. The CAM will be used to exclude delirium before each study visit and only if it indicates delirium would assessments be postponed by 2 weeks or as directed by the treating clinician.

#### Montreal Cognitive Assessment (MoCA)

The MoCA is a 30-point brief test of global cognitive function taking approximately 10 min to administer [31]. There are three alternate English language forms designed to minimise practice effects in longitudinal studies. The MoCA is included as the planned primary outcome in a future definitively powered clinical trial. A prior study validated the German language version of the MoCA in HD patients against a detailed neuropsychological battery of cognitive tests [42]. To our knowledge, this study will provide the first English language validation of the MoCA in HD patients against the Cogstate a detailed assessment of cognitive function. The MoCA is also available in Urdu and Bengali language, the two most common non-English native languages used by participants in the research sites.

#### Cogstate

The Cogstate system is a well validated computerised test that assesses a diverse range of key cognitive skills [32]. The Cogstate is available in 90 languages and uses multiple ‘parallel’ versions of the tests, thus minimising practice effects. The use of reliable repeated measures is of particular utility in studies in which participants may not be blind to their treatment status. The Cogstate system was also selected to reduce test fatigue and simplify test administration, whilst preserving strong test-retest reliability (rho = 0.81–0.89). It has the advantages of being portable, short (20–30 min), game-like in presentation and thus motivating, cross-culturally adaptable and language independent.

#### Quality of life

We will use the Assessment of Quality of Life (AQoL-6D) scale to measure patient’s quality of life [34]. This is a generic health-related quality of life instrument, which provides a profile relative to four life dimensions. Administration typically takes 5 to 10 min.

#### Activities of daily living

The Bristol Activity of Daily Living Scale will be used to measure activities of daily living in relation to cognitive impairment [35]. This is an informant-rated interview of 20 items each rated on 60-point scale. It was designed for use in patients with cognitive impairment.

#### Anxiety and depressive symptoms

The Hospital Anxiety and Depression Scale is a valid measure of anxiety and depression in patients with frequent hospital admissions where higher scores reflect greater depression and anxiety [37]. Systematic review identified a threshold score of 8 out of 21 for anxiety or depression [43] and 15 out of 21 as appropriate to refer patients into mental health care pathways.

#### Carer burden assessment

We will measure carer burden using Caregiver Burden scale, which was developed to assess perceived burden among caregivers of family members with cognitive impairment [36].

### Physiological measurements

#### Intradialytic hypotension

Intradialytic hypotension is an important secondary outcome for this trial as reducing these episodes are a plausible mechanism by which dialysate cooling might prevent cognitive decline. Intradialytic hypotension has been defined in several ways in prior studies making comparisons challenging. Symptoms are also infrequently self-reported by patients making symptom-based definitions problematic [44]. Recent data demonstrates that brain ischemia can occur at a variety of thresholds that would not typically be recognised as intradialytic hypotension [45]. A recent 77 patient study reported systolic blood pressure (BP) less than 100 mmHg or a 20% reduction in systolic BP from baseline as thresholds that maximised the probability of a nursing intervention rather than a session remaining asymptomatic [44]. Authors of a study of 11,801 HD patients reported that intradialytic hypotension defined as systolic BP less than 90 mmHg was potently associated with greater mortality [12] whilst definitions based on patient symptoms, nursing interventions or relative decreases in BP during dialysis were not. For this study, BP will be recorded as in routine clinical practice, before start and after end of dialysis session. In addition, BP will be checked every 30 min during HD treatment. For analysis, intradialytic hypotension will be defined as a fall in systolic BP during dialysis greater than 20% from baseline or absolute systolic BP less than 90 mmHg. Nursing interventions for intradialytic hypotension (slowing down ultrafiltration, giving additional fluid) are routinely recorded.

#### Intradialytic weight gain, ultrafiltration volume and ultrafiltration rate

Weight gain between dialysis sessions, the volume of fluid removed per treatment session (ultrafiltration volume) and the rate at which it is removed (ultrafiltration rate) will be extracted from routinely recorded HD treatment records and transferred monthly into the case report form by research staff after verification for any missing or implausible values. The feasibility of this process will be reported.

#### HD recovery time

The HD recovery time will be recorded by a simple question, “How long does it take you to recover from a dialysis session”. A mean of 3 reported recovery times across a dialysis week (Monday, Wednesday and Friday or Tuesday, Thursday and Saturday) will be assessed at baseline, 6 months and 12 months. Longer self-reported recovery time is independently associated with reduced health-related quality of life, increased hospitalisation and reduced survivals [38].

#### Adherence to treatment allocation

Adherence to the allocated dialysate temperature will be regularly checked and recorded by research staff at each research site using electronic records which enable distinction between prescribed and delivered dialysate temperature. Analysis would be by intention to treat.

### Outcome measures

#### Primary outcome measure

The primary outcome measure is change in cognition from baseline to 12 months, assessed by Montreal Cognitive Assessment (MoCA, v7.2) [31], in the standard and low temperature dialysis groups.

#### Secondary outcome measures

1. Frequency of intradialytic hypotension: to measure the frequency of intradialytic hypotension as an explanatory outcome
2. Recruitment rates: to measure recruitment to inform the design of a larger clinical trial
3. Attrition rates: to measure attrition rates to inform the design of a larger clinical trial
4. Non-recruitment reasons: to record reasons for non-recruitment and study attrition to inform the design of a larger clinical trial.
5. Depression rates: to measure depressive symptoms in the targeted population using the Hospital Anxiety and Depression Scale (HADS) [37].
6. Detailed assessment of cognition: to assess the acceptability and usability of a computerised cognitive assessment method (Cogstate) [32] for measuring cognition in dialysis patients, especially those from ethnic minorities. The Cogstate battery contains measures attention, psychomotor function, executive function and memory. The main outcome for this set of tests will be a composite cognitive score
7. To assess the burden of study-related interventions and assessments on carers using the Bristol Activities of Daily living scale and Carers Burden Assessment [36].
8. To assess the administration and suitability of the chosen method for measuring carers’ burden in this group.
9. To assess quality of life and activities of daily living in participants using the Assessment of Quality of Life (AQoL-6D) questionnaire [34].

### Data analysis

Data analysis will be performed by statisticians who are blinded to participant treatment allocation. A mixed method approach will be used, utilising semi-structured interviews, questionnaires and measurement of cognitive function. The 12 month follow-up period will be the key assessment time for all outcomes. Normally distributed data will be presented as mean (SD), skewed data as median (IQR) and categorical data as number (percent). The main aim of the qualitative component is to assess issues related to patient recruitment. This will include practicalities of implementing cooler dialysate, adherence to treatment, effectiveness of blindness process and identification of factors that may affect routine practice of treatment in various centres. We will apply thematic analysis to qualitative data collected from semi-structured interviews. Interviews will be on a 1:1 basis and will be audio-recorded. They will be transcribed by the research assistant and will be anonymised and securely stored, accessible only by the research team. The purpose of the quantitative analysis is to estimate the mean and standard deviation for MoCA at baseline and follow-up in both trial arms and obtain an estimate of the attrition. For all analysis, the level of significance will be set at 5%, so that 95% confidence intervals will be presented. This is a feasibility study and statistically or clinically significant changes in outcomes between groups are unlikely; hence, no between-group inferential comparisons will be made. However, a preliminary estimate of a treatment effect is relevant to sample size estimation of future definitive trials. As an exploratory analysis, we will conduct a complete case analysis of the primary and secondary outcomes. A linear regression model will be used for continuous outcomes (e.g. MoCA) and a logistic regression model will be used for binary outcomes. Each model will include the baseline measurement and treatment arm as independent variables. All patients randomised to their respective study arms will be included in the intention to treat analysis. Missing data, if any, will be managed using the last-observation carried forward approach. There will be no interim analysis of the data, though the safety data resulting from any SAE will be collated and made available to the Trial Steering Committee. All analysis will be conducted in Stata 15.

### Sample size

The outcome data from this feasibility study will be used to inform the sample size calculation for the definitive trial, by providing estimates of the primary outcome and its variability and the expected attrition. The study aims to recruit a total of 90 patients from four sites. Lancaster and co-workers outlined the key aspects of feasibility studies and indicated at least 30 patients per each arm are required to identify the sample variability (standard deviation) in key variables to enable the calculation of power for testing hypotheses in subsequent definitive studies [46]. The primary outcome in the definitive study is likely to be a value from the MoCA. With 45 patients in each arm, and if the mean (SD) value of the MoCA is 27 (2) in the control and intervention arms at the study start, we could expect a 95% confidence interval to range from 26.4 to 27.6 in each arm. This will give adequate precision for the estimate required in the study. With 45 patients in the control arm, and assuming a mean (SD) value of MoCA of 22 (3) after 12 months, we could expect a 95% confidence interval to range from 21.1 to 22.9. In the intervention arm, assuming a mean (SD) MoCA value of 25 (3) after 12 months, we could expect a 95% confidence interval to range from 24.1 to 25.9. Furthermore, with a total sample size of 90 patients, with an expected loss of 20% of the patients, a 95% confidence interval could be produced, ranging from 70.2 to 87.7%.

### Ethics

Ethical approval for trial has been obtained from the National Research Ethics Service Committee West Midlands-South Birmingham IRAS ID 234107 for all participating centres prior to study initiation and patient enrolment. The study will be performed in accordance with the Research Governance Framework, International Conference on Harmonisation Good Clinical Practice Guideline and the 2000 Scotland Revision of the Declaration of Helsinki. All participants are to provide written informed consent before any trial related procedure can occur. The University Hospitals Birmingham NHS Foundation Trust will provide trial oversight as the trial sponsor.

### Data management

All participants are assigned unique study numbers to ensure data is recorded in an anonymised fashion. All study documents are securely stored and only accessible to study staff and authorised personnel. All essential data transfer will happen within the secure networks.

### Study management

The study is monitored and audited by University Hospitals Birmingham NHS Foundation Trust under their remit as Sponsor and other regulatory bodies to ensure adherence to Good Clinical Practice and the UK Policy Framework for Health and Social Care Research. University Hospitals Birmingham NHS Foundation Trust holds standard NHS Hospital indemnity and insurance cover with NHS Litigation Authority for NHS Trusts in England, which apply to this study. The trial management committee (TMC) will meet at least quarterly during the duration of the study. They will provide guidance on the day to day running of the study, review study aims and ensure they are being met; they will report into the Trial Steering Committee (TSC). The TSC will be independent from the TMC, except for a sponsor representative. The TSC will meet at least every 6 months to review study data and offer guidance on the study outcomes and further direction of the potential full study. Supplementary Table 1 provides description of the roles for the study groups that are involved in the oversight and management/auditing of the trial.

### Protocol amendments

If any amendments to the study are required, the amendment will be agreed by the TMC and approved by the Sponsor. The appropriate approvals from the relevant regulatory authorities will be obtained and once received the amendment will be implemented. A full audit trail of the amendment will be contained in the Trial Master File.

### Harms

Serious adverse events (SAEs) will be reported using SAE reporting forms in the patient’s case report form. The principal investigator in each centre must report any SAEs to the Trial Co-ordinating Centre within 24 h of them becoming aware of it. The trial co-ordinator will liaise with the investigator to compile all the necessary information. The Trial Co-ordinating Centre is responsible for reporting adverse events to the sponsor and ethics committee within required timelines.

### Dissemination

The results from this study will be important for the kidney care community. The findings will be presented at national and international nephrology meetings. It is anticipated that this study will produce manuscripts suitable for submission to relevant peer-reviewed journals. The intention is to ensure all publications are open to access to encourage widespread dissemination of our findings.

### Patient and public involvement

Service users were important in designing the project and remain involved in its ongoing management. A service user representative is both a co-investigator and co-author to this research protocol and a service user group has led on key decisions around the frequency, setting and timing of assessments. Service users also helped write plain English summaries and gave feedback on the draft patient information sheets. Provision in the study design for use of language translators was led by service user. There is funding in the grant and local charitable funds will ensure the ongoing involvement of service users. Service users will also be invited to an end of study research event to share the results and future steps.

Supplementary Table S1 provides all of the items from the WHO trial registry. More details can be found within the body of the protocol in Additional file 4.

## Discussion

This feasibility trial is designed to inform the development of a definitive, fully powered, randomised, controlled clinical trial in the future. The main hypothesis that would be tested in this future trial is that patients treated with regular conventional haemodialysis will have a lesser decline in cognitive function and a better quality of life over one year by using cooler dialysis fluid at 35 °C, versus a standard dialysis fluid temperature of 36.5 °C. This also should reflect in improvements in their abilities for activities of daily living and therefore reduce carers’ burden. If successful, the treatment could be universally applied at no extra cost.

The main strengths of this study are (a) this is the first trial to assess the effect of cool dialysate temperature on cognitive function and quality of life for HD patients and carers, (b) the prospective multi-site, randomised, double-blinded, controlled trial design and (c) a range of outcomes will be assessed to inform study design of a future larger trial. This study will also allow validation of MOCA against a battery of computerised neuro-cognitive tests (Cogstate) in haemodialysis patients. The limitations of the study include patients requiring a good command of spoken English which may exclude those from an ethnic background and hence results may not be representative. Also, no comparisons will be made to other forms of renal replacement therapy (renal transplant and peritoneal dialysis) in terms of change in cognitive function.

One of the main practical issues we anticipate is difficulty in maintaining blinding of dialysate temperature to the patient, especially to patients who set up their own machines. Also, symptoms related to low temperature dialysis, feeling cold in the main, may also give it away. Secondly, recording BP every 30 min during HD treatment (lasting 4 h, 3 times a week) for 12 months may also prove challenging as this is not a routine practice in most dialysis centres in the UK; more so because these will be recorded by clinical nurses rather than research nurses. The third issue, which we think may make recruitment difficult, is the necessity for the patient to attend research appointments on a non-dialysis day. Although they will need to attend only 3 times over 12-month period, haemodialysis patients are generally not keen to attend hospital appointments outside their dialysis sessions. However, how these issues are dealt with during conduct of this study will help inform the design of the future substantive trial.

Supplementary Table S1 provides all of the items from the WHO trial registry. More details can be found within the body of the protocol in Additional file 4.

## Trial status

Protocol version 2.16, dated 21 June 2018

Date recruitment began: 20 December 2017

Approximate date the recruitment will be completed: 31 October 2020

## Supplementary information

**Additional file 1.** Patient Information pack.

**Additional file 2.** Consent Form.

**Additional file 3: Supplementary Table S1**- WHO Trial registry dataset.

**Additional file 4.**

## Data Availability

Not applicable to this publication. Following completion of the trial and publication of the primary analyses, data will be made available to investigators under the conditions of a data sharing agreement. This will include group- and individual-level fully anonymized data.

## Abbreviations

ADL: Activities of Daily Living
AQoL: Assessment of Quality of Life
CAM: Confusion Assessment Method
CKD: Chronic kidney disease
ESKD: End stage kidney disease
HADS: Hospital Anxiety and Depression Scale
HD: Haemodialysis
MoCA: Montreal Cognitive Assessment
NHS: National Health Service
SD: Standard deviation
SAE: Serious adverse events
TSC: Trial Steering Committee
