# Supplementary material for: Evaluation of the effect of Cooled HaEmodialysis on Cognitive function in patients suffering with end-stage KidnEy Disease (E-CHECKED): feasibility randomised control trial protocol
Source: Trials. 2020 Sep 30;21:820. doi: 10.1186/s13063-020-04725-0 (PMC7526411; doi:10.1186/s13063-020-04725-0)
Supplement: Supplementary file 1 — Additional file 1. Patient Information pack. [file 13063_2020_4725_MOESM1_ESM.doc]

**Evaluation of the Effect of Cooled Haemodialysis on Cognitive Function in Patients suffering with End-stage Kidney Disease: Feasibility study (E-CHECKED)**

**Patient Information Sheet**

**Invitation**

We would like to invite you to take part in our research study. Before you decide we would like you to understand why the research is being done and what it would involve for you. One of our team will go through the information sheet with you and answer any questions you have.

Talk to others about the study if you wish. This information sheet tells you the purpose of this study and what happens if you decide to take part. Please ask any questions if there is anything that is not clear.

**What is the purpose of the study?**

Patients with kidneys that do not work properly need a kidney machine to remove excess fluid and waste products from the body; a process called haemodialysis. Haemodialysis is a huge burden for patients and their family or carers. Some patients may experience unpleasant symptoms such as poor quality-of-life with some periods of depression, thinking difficulties and memory problems (known as cognitive impairment). This reduces the ability to cope with taking many medicines, eating and drinking restrictions. Dialysis patients value quality-of-life over life expectancy, but no treatments have been shown to help.

During haemodialysis, fluids are removed that cause sudden drops in blood pressure, with dizziness and muscle cramps. These drops in blood pressure damage the heart and brain and may cause some cognitive impairment in haemodialysis patients. Several, small, short-term studies suggest using cooler fluid during haemodialysis reduces blood pressure drops. A recent small trial showed cooler dialysis fluid might prevent brain injury by reducing these drops in blood pressure. Few studies report how well tolerated cooler dialysis fluid is, but effects on cognitive impairment and quality-of-life are not known. As all patients can use cooler dialysis fluid at no added cost, a trial to test the health benefits to prevent cognitive impairment and preserve quality-of-life is urgently needed.

This study will randomly give 90 haemodialysis patients either standard or cooled fluid for one year. This study is a useful way to let us test the possible issues with how to run the next study that will be big enough to determine this.

**Why have I been invited?**

You have been invited to take part in the study as you are receiving haemodialysis.

**Do I have to take part?**

It is up to you to decide to join the study. We will describe the study and go through this information sheet. If you agree to take part, we will then ask you to sign a consent form. You are free to withdraw at any time, without giving a reason. This would not affect the standard of care you receive.

**What will happen to me if I take part?**

Sometimes we don‘t know which way of treating patients is best. To find out, we need to compare different treatments in this study standard dialysis with dialysis using a cooler dialysis fluid.

We put people into groups and give each group a different treatment. The results are compared to see if one is better. To try to make sure the groups are the same to start with, each patient is put into a group by chance (randomly).

If you decide to take part in this study you will be randomised to either receive dialysis as you normally would or to receive dialysis at a cooler temperature for the next 12 months. The process of dialysis will be the same and will take the same amount of time. A computer programme will decide which dialysis you will have, the research team will not be able to alter or affect this.

You will attend your dialysis as normal, if randomised to the cool dialysis treatment the dialysis unit will arrange for this to happen, during your normal dialysis. During dialysis you will be closely monitored, as you would normally.

The research team will ask to see you at the beginning of the study, 6 and 12 months and ask you to complete some questionnaires and cognitive tests. We will also ask you to refer a nominated person and or carer who is close to you who can complete two questionnaires at the beginning of the study, 6 and 12 months. You will be able to take part in the study even if your carer declines to participate.

**What will I have to do?**

You will have to attend your dialysis sessions, as you would normally. In addition to this the research team will meet with you at two additional time points, arranged to suit you, at 6 and 12 months. They will ask you to complete the cognitive tests and some short questionnaires.

**What are the alternatives for diagnosis or treatment?**

An alternative to this research study is to continue having your current care.

**What are the possible disadvantages and risks of taking part?**

If you were to receive the cooled dialysis temperature you may feel cold, however you will be allowed to bring your own blankets or extra clothing to keep yourself warm.

A disadvantage could be that you have to give up time to complete a series of questionnaires and cognitive tests. However this will only be at the start of the study, 6months and at twelve months.

There is a chance that through the study we may identify that you have some early cognitive impairment. This information will be communicated to you and your GP and any relevant referrals will be made. The same is true with any other findings discovered during the study which may require further care outside of your direct care team.

**What are the side effects of any treatment received when taking part?**

Potential side effects would be similar to those that you would possibly already experience during your routine haemodialysis. Additionally, if you were to receive the cooled dialysis temperature you may feel cold, however you will be allowed to bring your own blankets or extra clothing to keep yourself warm.

**What are the possible benefits if taking part?**

We cannot promise the study will help you but the information we get from this study will help improve the treatment of people.

**What happens when the research stops?**

If you are selected to receive the cooled dialysis, and it is successful, this will be provided to you as long as it continues to work for you, and your treating clinician agrees.

We will also be happy to share the results with you if you wish. Once the study has finished, your data will be kept secure at Birmingham Heartlands Hospital for five years.

**What if there is a problem?**

You will be compensated for negligent harm according to NHS indemnity guidelines. If you wish to complain about any aspect of the way you have been approached or treated during the course of this study, the normal National Health Service complaints mechanisms will be available to you. Copies of these guidelines are available on request.

If you wish to complain about how you have been treated during this study please contact Patient Advice and Liaison Service (PALS)

[www.heartlands-ppi.co.uk/pals.asp](http://www.heartlands-ppi.co.uk/pals.asp)

**Phone (0121) 424 1212**

Or email [pals@heartofengland.nhs.uk](http://www.heartlands-ppi.co.uk/contact.asp?recipient=1#email)

If for some reason you lose capacity to undertake the study, you and your data will be withdrawn and you will no longer be taking part in the research.

**Will my taking part in the study be kept confidential?**

Yes. If you join the study, some parts of your medical records and the data collected for the study will be looked at by authorised persons from the Trust for the research. They may also be looked at by authorised people to check that the study is being carried out correctly. All will have a duty of confidentiality to you as a research participant and we will do our best to meet this duty.

The data collected will be used to answer this piece of research. The data will be accessible to your direct care team and the research team, when required. It will be collected via your medical records and from the information you provide during your visits. Your data will be kept safe via University Hospital Birmingham’s secure computer network and any physical information will be kept in a secure location at Birmingham Heartlands Hospital.

**What if relevant new information becomes available?**

Sometimes we get new information about the treatment being studied. If this happens, your research doctor will tell you and discuss whether you should continue in the study. If you decide not to carry on, your research doctor will make arrangements for your care to continue. If you decide to continue in the study he may ask you to sign an agreement outlining the discussion.

**What will happen if I don’t want to carry on with the study?**

You are free to withdraw at any time from the study without giving any reason. If you do decide to withdraw, the person who you refer as your nominated person will also have to be withdrawn from the study.

**Involvement of the General Practitioner/Family doctor (GP)**

We will notify your GP so that they are aware of your involvement in the study.

**What will happen to any samples I give?**

The only samples that will be collected are part of your routine haemodialysis treatment.

Routine blood samples that are analysed will not be stored for research purposes and will be destroyed in accordance with University Hospitals Birmingham NHS Foundation Trust’s Laboratory standard operating procedures.

**What will happen to the results of the research study?**

The data may be published in peer reviewed journals and presented at meetings. No individual will be identified in any publication. If you want a copy of the results, this can be sent to you if you ask the research team. To do this, your contact details will be kept in a secure location, accessible by the study team only, in order to send you this information once the study has finished.

**Who is organising and funding the research?**

The research is funded by the National Institute for Health Research (NIHR) Research for Patient Benefit (RfPB) and is Sponsored by the University Hospitals Birmingham NHS Foundation Trust.

**Who has reviewed the study?**

All research in the NHS is looked at by independent group of people, called a Research Ethics Committee, to protect your interests. This study has been reviewed and given favourable opinion by Research Ethics Committee and Health Research Authority.

**Further information and contact details**

If you would like to ask any questions please contact:

Professor George Tadros on 01214240247
