# Supplementary material for: Evaluation of the effect of Cooled HaEmodialysis on Cognitive function in patients suffering with end-stage KidnEy Disease (E-CHECKED): feasibility randomised control trial protocol
Source: Trials. 2020 Sep 30;21:820. doi: 10.1186/s13063-020-04725-0 (PMC7526411; doi:10.1186/s13063-020-04725-0)
Supplement: Supplementary file 2 — Additional file 2. Consent Form. [file 13063_2020_4725_MOESM2_ESM.pdf]

IRAS ID 234107

Centre Name:

Study Number:

PATIENT CONSENT FORM

Title of Project: Evaluation of the Effect of Cooled Haemodialysis on Cognitive Function in Patients suffering with End-stage Kidney Disease: Feasibility study (E-CHECKED)

Principal Investigator: **Professor George Tadros**

Please initial all boxes

1. I confirm that I have read and understand the information sheet dated_________ (version________) for the above study. I have had the opportunity to consider the information, ask questions and have had these answered satisfactorily. ☐

2. I understand that my participation is voluntary and that I am free to withdraw at any time without giving any reason, without my medical care or legal rights being affected. ☐

3. I understand that relevant sections of my medical notes and data collected during the study, may be looked at by individuals, from regulatory authorities or from the NHS Trust, where it is relevant to my taking part in this research. I give permission for these individuals to have access to my records. ☐

4. I understand that I am taking part in a study whereby I may randomised to either receive my standard dialysate temperature, or receive a reduced dialysate temperature, reduced down over six weeks, with the aim to reach 35 Degrees centigrade, if tolerated. As the patient you will be blinded to the temperature which means that you are not able to ask and we request that you do not try to see what temperature you are randomised to. ☐

5. I agree to my GP being informed of my participation in the study. ☐

6. I agree to take part in the above study. ☐

Name of Participant | Date | Signature

Please provide a phone number of the Nominated person/carer you wish to invite to the study.

____

Name of Person taking consent. | Date | Signature

When completed: file in the researcher file, copy in medical notes, copy in renal dialysis notes and copy to participant

University Hospitals Birmingham
NHS Foundation Trust

IRAS ID 234107

Centre Name:

Study Number:

CARER CONSENT FORM

Title of Project: Evaluation of the Effect of Cooled Haemodialysis on Cognitive Function in Patients suffering with End-stage Kidney Disease: Feasibility study (E-CHECKED)

Principal Investigator: **Professor George Tadros**

Please initial all boxes

1. I confirm that I have read and understand the information sheet dated ____________ (version___________) for the above study. I have had the opportunity to consider the information, ask questions and have had these answered satisfactorily. ☐

2. I am aware my participation is entirely voluntary. I may choose not to be in the study or to leave the study at any time by telling a member of the study team. I understand that if I leave the study, then the subject with whom I am participating in the study will still be able to continue. I will not be penalised in any way. ☐

3. I understand that data collected during the study may be looked at by individuals, from regulatory authorities or from the NHS Trust, where it is relevant to my taking part in this research. I give permission for these individuals to have access to my records. ☐

4. I consent to enter this study as a Carer/nominated individual. ☐

Name of Participant	Date	Signature
Name of Person taking consent.	Date	Signature

When completed: file in the researcher file and copy to participant.
