# Supplementary material for: Evaluation of the effect of Cooled HaEmodialysis on Cognitive function in patients suffering with end-stage KidnEy Disease (E-CHECKED): feasibility randomised control trial protocol
Source: Trials. 2020 Sep 30;21:820. doi: 10.1186/s13063-020-04725-0 (PMC7526411; doi:10.1186/s13063-020-04725-0)
Supplement: Supplementary file 3 — Additional file 3: Supplementary Table S1- WHO Trial registry dataset. [file 13063_2020_4725_MOESM3_ESM.doc]

**Supplementary Table S1- WHO Trial registry dataset**

| **Data category** | **Information** |
| --- | --- |
| Primary registry and trial identifying number | ClinicalTrials.gov NCT03645733 |
| Date of registration in primary registry | 24/08/2018 |
| Secondary identifying numbers | IRAS ID 234107, Sponsor reference 2017054MH |
| Source(s) of monetary or material support | National Institute for Health Research. Research for Patient Benefit Grant (Reference PB-PG-1215-20008) |
| Primary sponsor | Dr Sarah Pountain, University Hospitals Birmingham NHS Foundation Trust, Birmingham Heartlands Hospital, Bordesley Green East, Birmingham B9 5SS. Tel: 0121 424 3631. E-mail sarah.pountain@heartofengland.nhs.uk |
| Secondary sponsor(s) | None |
| Contact for public queries | Professor George Tadros, University Hospitals Birmingham NHS Foundation Trust, Birmingham Heartlands Hospital, Bordesley Green East, Birmingham B9 5SS. Tel: 0121 424 0247. E-mail George.tadros@nhs.net |
| Contact for scientific queries | Professor George Tadros, University Hospitals Birmingham NHS Foundation Trust, Birmingham Heartlands Hospital, Bordesley Green East, Birmingham B9 5SS. Tel: 0121 424 0247. E-mail George.tadros@nhs.net |
| Public title | Evaluation of the Effect of Cooled Haemodialysis on cognitive Function in Patients suffering with End-stage Kidney Disease: Feasibility Study |
| Scientific title | Evaluation of the Effect of Cooled Haemodialysis on cognitive Function in Patients suffering with End-stage Kidney Disease: Feasibility Study |
| Countries of recruitment | United Kingdom |
| Health condition(s) or problem(s) studied | Cognitive function |
| Intervention(s) | Standard treatment: dialysis fluid temperature of 36.5°C |
|  | Active treatment: dialysis fluid temperature of 35.0°C |
| Key inclusion and exclusion criteria | Ages eligible for study: ≥18 years Sexes eligible for study: both Accepts healthy volunteers: no  Inclusion criteria:  1 Aged 18 years or greater  2 Receiving HD three times per week for ESKD, for at least 3 months  3 Having proven mental capacity to understand the study and give informed consent  Exclusion criteria:  1 Established diagnosis of dementia in a memory clinic or specialised service  2 Receiving Acetylcholine Esterase Inhibitors  3 Receiving antipsychotic or antidepressants unless stable on treatment for at least 6 weeks  4 Current participation in a study of an investigational medicinal product  5 Inter-current infection  6 An operation date for a living donor kidney transplant within the period of the trial  7 Patients expected to survive less than 1 year according to the treating nephrologist  8 Patients prone to intra-dialytic hypotension or cardiovascular instability during HD according to the treating nephrologist  9 Patients who are currently taking triptans, dopamine antagonists, tramadol, sedative and opioid analgesics  10 Patients who have a known diagnosis or have other psychiatric conditions, including severe depression, bipolar affective disorder, severe anxiety, panic disorder, substance misuse or psychosis  11 Currently involved in another intervention study |
| Study type | multi-site, prospective, randomised, double-blinded, controlled, feasibility trial |
|  | Primary purpose: test feasibility of definitive intervention trial |
| Date of first enrolment | 20/12/2017 |
| Target sample size | 90 |
| Recruitment status | Recruiting |
| Primary outcome(s) | Change in cognition from baseline to 12 months, assessed by Montreal Cognitive Assessment (MoCA, v7.2) in the standard and low temperature dialysis groups |
| Key secondary outcomes | 1 Frequency of intradialytic hypotension: to measure the frequency of intradialytic hypotension as an explanatory outcome  2 Recruitment rates: to measure recruitment to inform the design of a larger clinical trial  3 Attrition Rates: to measure attrition rates to inform the design of a larger clinical trial  4 Non-recruitment reasons: to record reasons for non-recruitment and study attrition to inform the design of a larger clinical trial  5 Depression rates: to measure depressive symptoms in the targeted population using the Hospital Anxiety and Depression Scale (HADS)  6 Detailed assessment of cognition: to assess the acceptability and usability of a computerised cognitive assessment method (Cogstate) for measuring cognition in dialysis patients, especially those from ethnic minorities The Cogstate battery contains measures of attention, psychomotor function, executive function and memory the main outcome for this set of tests will be a composite cognitive score  7 To assess the burden of study-related interventions and assessments on carers using the Bristol Activities of Daily living scale and Carers Burden Assessment  8 To assess the administration and suitability of the chosen method for measuring carers' burden in this group  9 To assess quality of life and activities of daily living in participants using the Assessment of Quality of Life (AQoL-6D) questionnaire |
| Trial Management Committee | Professor George Tadros  Professor Indranil Dasgupta  Professor Neil Thomas  Professor Gavin Woodhall  Professor Helen Griffiths  Dr Aghogho Odudu  Dr Jyoti Baharani  Dr Niall Fergusson  Mr Samir Youseff  Professor Paul Maruff  Professor John Harrison |
| Trial Steering Committee | Prof Asif Ahmed (Dean of Aston Medical School)  Prof Alex Cappello (Prof of Psychology, University of Birmingham)  Dr Atef Michael (Consultant in Geritaric Medicine, Russell’s Hall Hospital, Dudley)  Prof Martin O’Rell (Nottingham)  Sponsor Representative from University Hospitals Birmingham NHS Foundation Trust  Dr Mark Temple (Consultant in Renal Medicine, University Hospitals Birmingham NHS Foundation Trust)  Dr Farooq Khan (Consultant Psychiatrist for the elderly, Birmingham and Solihull Mental Health Foundation Trust)  Carol Holland (Aston University)  James Martin (Statistician, University of Birmingham)  Renal research nurse from Renal Unit, University Hospitals Birmingham NHS Foundation Trust |
