# Supplementary material for: Evaluation of the effect of Cooled HaEmodialysis on Cognitive function in patients suffering with end-stage KidnEy Disease (E-CHECKED): feasibility randomised control trial protocol
Source: Trials. 2020 Sep 30;21:820. doi: 10.1186/s13063-020-04725-0 (PMC7526411; doi:10.1186/s13063-020-04725-0)
Supplement: Supplementary file 4 — Additional file 4. [file 13063_2020_4725_MOESM4_ESM.pdf]

University Hospitals Birmingham
NHS Foundation Trust

Evaluation of the Effect of Cooled Haemodialysis on cognitive Function in Patients suffering with End-stage Kidney Disease: Feasibility Study (E-CHECKED)

This protocol has regard for the HRA guidance and order of content.

RESEARCH REFERENCE NUMBERS

**IRAS Number:**	234107
**SPONSORS Number:**	2017054MH
**FUNDERS Number:**	Research for Patients Benefit (RfPB) PB-PG-1215-20008

SIGNATURE PAGE

The undersigned confirm that the following protocol has been agreed and accepted and that the Chief Investigator agrees to conduct the study in compliance with the approved protocol and will adhere to the principles outlined in the Declaration of Helsinki, the Sponsor's SOPs, and other regulatory requirement.

I agree to ensure that the confidential information contained in this document will not be used for any other purpose other than the evaluation or conduct of the investigation without the prior written consent of the Sponsor

I also confirm that I will make the findings of the study publically available through publication or other dissemination tools without any unnecessary delay and that an honest accurate and transparent account of the study will be given; and that any discrepancies from the study as planned in this protocol will be explained.

For and on behalf of the Study Sponsor:

Signature: Date: ....../....../......

....

Name (please print):

....

Position:
....

Chief Investigator:

Signature: Date:
..../....../......

Name: (please print):

....

KEY STUDY CONTACTS

Chief Investigator	Professor George Tadros,<br><br>University Hospitals Birmingham NHS Foundation Trust, Birmingham Heartlands Hospital, Bordesley Green East, Birmingham B9 5SS. Tel: 0121 424 0247. E-mail George.tadros@nhs.net
Sponsor	University Hospitals Birmingham NHS Foundation Trust, Birmingham Heartlands Hospital, Bordesley Green East, Birmingham B9 5SS.
Funder	NIHR Research for Patient Benefit (RfPB), ref: PB-PG-1215-20008
Committees	Trial Management Committee and Trial Steering Committee

STUDY SUMMARY

Study Title	Evaluation of the Effect of Cooled Haemodialysis on cognitive Function in Patients suffering with End-stage Kidney Disease: Feasibility Study
Short title	E-CHECKED
Study Design	Multi -site prospective, randomised, double-blinded, controlled, feasibility trial
Study Participants	Patients receiving haemodialysis three-times a week for End-Stage Kidney Disease (ESKD) for at least 3 months in specialised sites.
Planned Size of Sample (if applicable)	90 patients (45 patients in the intervention group and 45 patients in the control group).
Follow up duration (if applicable)	12 months
Research Question/Aim(s)	**Aims:**<br>We aim to perform a feasibility study that will inform the development of a definitive, fully powered, randomised, controlled clinical trial in the future. The main hypothesis that would be tested in this future trial is that patients treated with regular conventional haemodialysis will have a lesser decline in cognitive function and a better quality of life over one year by using cooler dialysis fluid at 35°C, versus a standard dialysis fluid temperature of 36.5°C. This also should reflect in improvements in their abilities for activities of daily living and therefore, reduce carers' burden. If successful the treatment could be universally applied at *no extra cost.*<br><br>**Primary objective:**<br>To test the feasibility of using lower temperature dialysis fluid in preventing the decline in cognitive function and improve the quality of life in haemodialysis patients.<br><br>**Secondary objectives:**<br>1) To provide an estimation of the variability in the outcome measures for the cooled dialysis and standard treatment arms, to inform a future, adequately powered, definitive trial.<br>2) To measure the frequency of intradialytic hypotension as an explanatory outcome.<br>3) To measure recruitment and attrition rates to inform the design of a larger clinical trial.<br>4) To record reasons for non-recruitment and study attrition to inform the design of a larger clinical trial.

	5) To measure depression in targeted population to be able to estimate exclusion rates of patients who would be suffering from “Depressive Pseudo Cognitive Impairment” from the future trial.<br>6) To refine the monitoring process for safety and any unexpected untoward events.<br>7) To assess the burden of study-related interventions and assessments on patients and carers.<br>8) To assess the administration, suitability and adherence of the chosen cognitive method for patients, especially those from ethnic minorities.<br>9) To assess the administration and suitability of the chosen scales for quality of life measures and activities of daily living in haemodialysis participants.<br>10) To assess the administration and suitability of the chosen method for measuring carers’ burden in this group.

Trial Management Committee

Professor George Tadros
Professor Neil Thomas
Professor Gavin Woodhall
Professor Helen Griffiths
Dr Aghogho Odudu
Dr Jyoti Baharani
Dr Niall Fergusson
Samir Youseff
Professor Paul Maruff
Professor John Harrison
Dr Indranil Dasgupta

Trial Steering Committee

Prof Asif Ahmed (Dean of Aston Medical School)
Prof Alex Cappello (Prof of Psychology, University of Birmingham)
Dr Atef Michael (Consultant in Geritaric Medicine, Russell's Hall Hospital, Dudley)
Prof Martin O'Rell (Nottingham)
Sponsor Representative from University Hospitals Birmingham NHS Foundation Trust
Dr Mark Temple (Consultant in Renal Medicine, University Hospitals Birmingham NHS Foundation Trust)
Dr Farooq Khan (Consultant Psychiatrist for the elderly, Birmingham and Solihull Mental Health Foundation Trust)
Carol Holland (Aston University)
James Martin (Statistician, University of Birmingham)
Renal research nurse from Renal Unit, University Hospitals Birmingham NHS Foundation Trust

LIST OF CONTENTS

KEY STUDY CONTACTS..3

LIST OF CONTENTS 7

STUDY FLOW CHART..10

1. BACKGROUND AND RATIONALE 11

2. RESEARCH QUESTION/AIM(S) 13
2.1 Objectives 13
2.2 Outcome 14

3. STUDY DESIGN, METHODS OF DATA COLLECTION AND DATA ANALYIS ..14

4. STUDY SETTING 15

5. STUDY POPULATION AND RECRUITMENT 15
5.1 Study population 15
5.2 Study Interventions 15
5.3 Recruitment.. 16

5.4 ELIGIBILITY CRITERIA...17
5.4.1 Inclusion criteria.. 17
5.4.2 Exclusion criteria 17
5.4.3 Inclusion Criteria (Carers) 18
5.4.4 Exclusion Criteria (Carers)... 18
5.5 Participant Withdrawal 18
5.6 Randomisation 18

6. DATA COLLECTION 18
6.1 Baseline Data Collection... 18
6.2 Follow up Data collection 19

6.3 DATA MANAGEMENT AND ANALYSIS 24
6.3.1 Sample Size 24
6.3.2 Data management 24
6.3.3 Data analysis 24

7. SAFETY & ADVERSE EVENT MANAGEMENT 26
7.1 Definitions 26
7.1.1 Adverse Events (AE) 26
7.1.2 Serious Adverse Events (SAEs) 26
7.1.3 Serious Adverse Reactions (SARs) 26
7.2 Reporting Procedures 26
7.2.1 Causality 26
7.2.2 Reporting ARs 27
7.2.3 Reporting SAEs, SAR's and SUSARs 27

8. AUDIT & MONITORING 27

9. STUDY MANAGEMENT 28

10. REGULATORY APPROVALS 28
10.1 Sponsorship and Indemnity 28

11. PATIENT AND PUBLIC INVOLVEMENT 28

12. PROTOCOL COMPLIANCE 29

13. AMENDMENTS 29

14. DISSEMINATION POLICY 29
14.1 Authorship eligibility guidelines and any intended use of professional writers 29

15 REFERENCES 30

STUDY FLOW CHART

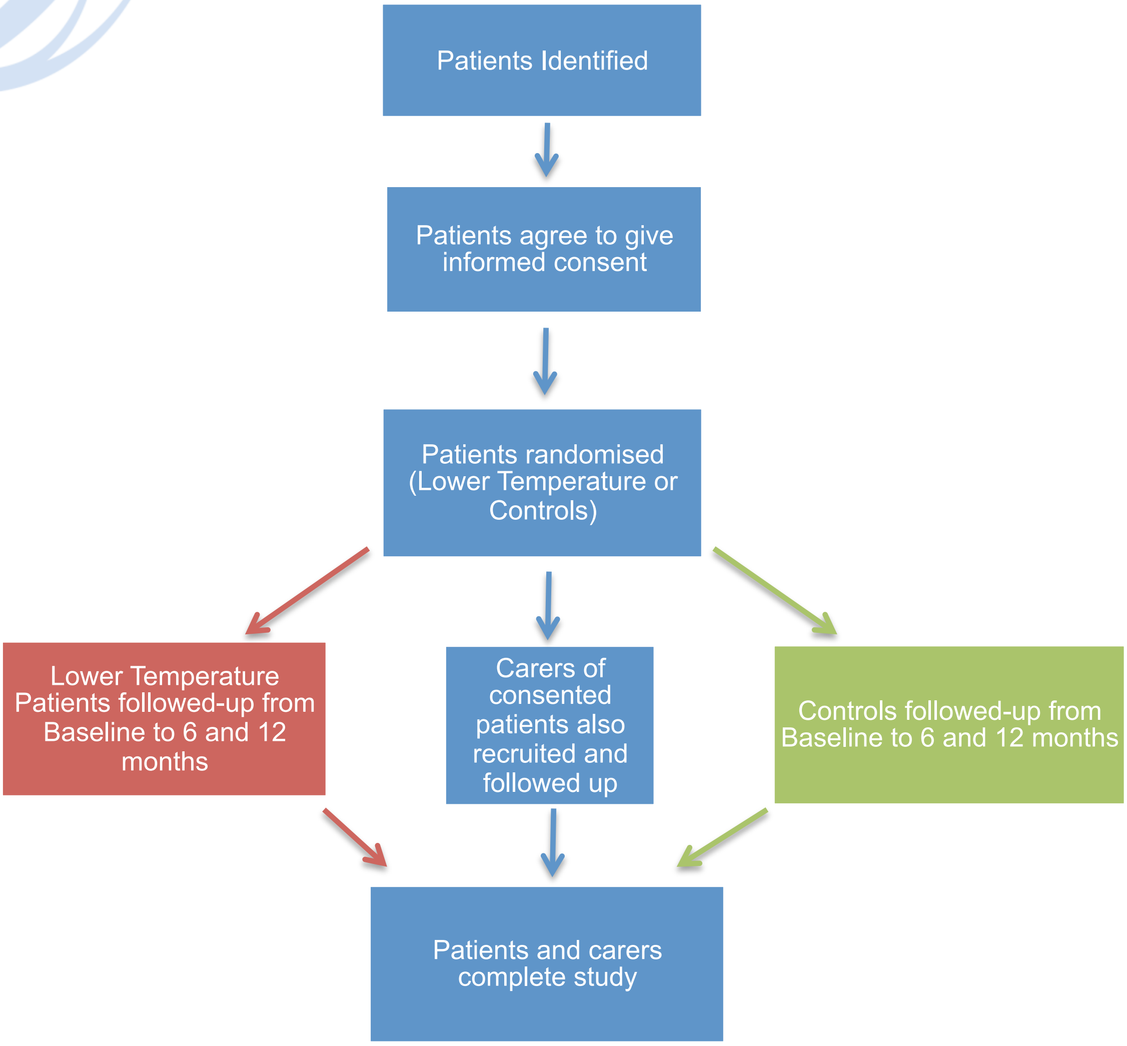

STUDY PROTOCOL

Evaluation of the Effect of Cooled Haemodialysis on cognitive Function in Patients suffering with End-stage Kidney Disease: Feasibility Study (E-CHECKED)

1. BACKGROUND AND RATIONALE

Patients receiving dialysis have poor life expectancy, reduced quality of life, frequent depression and cognitive impairment

Patients with End-Stage Kidney Disease (ESKD) need haemodialysis to remove excess toxins and fluid from the body and maintain life. They also must restrict their fluid intake, take a median of 19 medications and follow a special diet[1]. In the UK, 26 000 patients receive haemodialysis at a hospital three times a week for around 4 hours at a yearly cost of £636 million. The numbers needing haemodialysis are rising by 7% per year due to an increase in ageing, diabetes, obesity and hypertension. The best form of treatment for kidney failure is kidney transplantation, but there is a shortage of organ donors with older people being least likely to receive a kidney transplant. The average age of dialysis patients in the UK is 65 with 4-year survival expectancy less than 40% - which is worse than for most cancers[2]. The three most common causes of death are cardiovascular disease, infections and cancer[2] with the greatest mortality in the first three months of starting dialysis [3]. Haemodialysis is a huge burden for patients and their family or carers [1]. Most endure unpleasant dialysis-related symptoms and reduced quality-of-life with high rates of depression, cognitive impairment, hospital admissions and social isolation [4-7]. Unsurprisingly, dialysis patients value quality-of-life more than life expectancy [8,9]. Several medications currently used at considerable cost to improve survival and quality-of-life have shown no benefit [10-12].

High rates of cognitive impairment in dialysis patients are poorly understood

Increasing severity of Chronic Kidney Disease (CKD) is associated with a graded increase in prevalence of cognitive impairment[13,14] and decrease in brain perfusion independent of vascular risk factors[15]. Diagnostic methods vary but recent reviews summarise at least moderate cognitive impairment in 30-70% of dialysis patients[6,16,17]. Cognitive impairment in haemodialysis patients is independently associated with higher rates of depression and mortality[6,18]. To date, no interventions are proven to slow cognitive decline and this poorly understood association was recently reviewed[19]. Co-segregation of atherosclerotic risk factors[18], cannot entirely account for excess risk[20]. There are multiple factors CKD and haemodialysis specific factors including oxidative stress, malnutrition and inflammation[19]. Haemodialysis allows accumulation of several neurotoxins[21] that reduce brain perfusion and blood-brain barrier integrity[22].

Intradialytic hypotension is implicated in excessive cognitive impairment

Haemodialysis involves cycles of removing varying volumes of fluid, electrolytes and toxins that accumulate between treatments. Hypotension partially results from fluid removal rates exceeding plasma refill rates. Ubiquitous left ventricular hypertrophy and aortic stiffness further lower the threshold for haemodialysis to inflict recurrent multi-organ ischemia-reperfusion injury[23]. Haemodialysis might cause worsening of cognitive impairment by inducing haemodynamic instability, fluid shifts, cerebral ischaemia or cerebral oedema[19,23-25]. Intradialytic hypotension is common affecting

30-40% of treatments and is consistently associated with at least a 30% increase in mortality and reduced quality-of-life [26]. These dynamic changes in Blood Pressure (BP) and perfusion might be associated with altered cognition but the data are sparse and conflicting, possibly reflecting differences in study design; such as different methods and timings for cognitive assessments. Several small studies show cognitive function is best immediately before haemodialysis, worse during haemodialysis and improves the day after with a possible link to sudden fluid removal [27]. Our own experience, using the Montreal Cognitive Assessment in 100 haemodialysis patients also showed cognitive decline during haemodialysis. A recent retrospective study of 121,000 patients report that peritoneal dialysis is associated with a 26% lesser-adjusted risk of newly diagnosed dementia compared to haemodialysis [28]. One plausible mechanism of that benefit is that peritoneal dialysis does not cause sudden reductions in blood pressure [29].

Low temperature dialysis prevents intradialytic hypotension and may protect the heart and brain from ischemia

Absence of intradialytic hypotension is emerging as a novel treatment goal [30]. One possible way to prevent hypotension is to increase treatment time or frequency to allow more gentle fluid removal. A clinical trial of 245 patients showed 6 times weekly haemodialysis improved physical health scores whilst reducing intradialytic hypotension, fluid gains and left ventricular mass [31]. A preliminary repeated measures study of 12 patients showed extended overnight haemodialysis was associated with improved cognitive function scores [32]. These data are encouraging but come at the expense of increased treatment complications, cost and are currently unfeasible in most UK centres and worldwide. The use of cooler dialysate (34-35°C) to prevent intradialytic hypotension was first described in 1981[33]. However, this therapy remains greatly underused because of perceptions about thermal symptoms [34,35]. Cooler dialysate doesn’t necessarily lower core-temperature and it is thought to prevent intradialytic hypotension by preventing a rise in core temperature and subsequent systemic vasodilation36. A recent systematic review of cooler dialysate analyzed 26 trials in 484 patients [37]. Compared with standard temperature dialysis, cooler dialysis reduced the rate of intradialytic hypotension by 70% (95% CI, 49-89%). Confidence in the estimates was limited by small sample sizes, attrition and a lack of appropriate blinding with no trial reporting long-term outcomes [37]. A recent RfPB grant funded pilot clinical trial in 38 patients, showed lower temperature of dialysis fluid prevented the progression of ischemic brain white matter changes after one year which appeared to be linked to hemodynamic stability [38,39]. The same trial also reported cooler dialysis fluid improved cardiac structure and function [40]. The effects of cooler dialysate on cognitive impairment, quality-of-life and illness burden have not been robustly tested or are not known. How well tolerated cooler dialysis fluid is also not well reported. A recent editorial called for larger trials using this cheap and universally applicable intervention that focused on these patient important outcomes [34,35]. The current low usage of cooler dialysate in the UK affords an opportunity to definitively test this simple modification to haemodialysis as a potential intervention to prevent cognitive dysfunction and quality-of-life. There are several uncertainties around study design of a definitive trial of cooler dialysate and cognitive impairment, hence the need to formally assess these in a feasibility study.

2. RESEARCH QUESTION/AIM(S)

We aim to perform a feasibility study that will inform the development of a definitive, fully powered, randomised, controlled clinical trial in two years. The main hypothesis that would be tested in this future trial is that patients treated with regular conventional haemodialysis will have a lesser decline in cognitive function and a better quality of life over one year by using cooler dialysis fluid at 35°C, versus a standard dialysis fluid temperature of 36.5°C. This also should reflect in bettering their abilities for activities of daily living and therefore, reduce carers' burden. If successful the treatment could be universally applied at *no extra cost.*

This study will inform the design of a definitive randomised controlled clinical trial that would examine the efficacy of cooler dialysis fluid in reducing cognitive decline in patients receiving haemodialysis for End-Stage Kidney Disease (ESKD). Cognitive decline has a serious negative effect on patients' quality of life, mood and ability to perform regular activities of daily living. Haemodialysis patients are a high-risk population with poor outcomes that lead to significant health and social care costs and increased disease burden. There is significant need for interventions that would reduce the burden of disease, especially as developing cognitive impairment or dementia is one of the most feared health outcomes by patients and the most costly in terms of long term service provision.

2.1 Objectives

Primary objective:
To test the feasibility of the investigation of lower temperatures of dialysis fluid in preventing the decline in cognitive function and improve the quality of life in haemodialysis patients.

Secondary objectives:
1) To provide an estimation of the variability in the outcome measures for the cooled dialysis and standard treatment arms, to inform a future, adequately powered, definitive trial.
2) To measure the frequency of intradialytic hypotension as an explanatory outcome.
3) To measure recruitment and attrition rates to inform the design of a larger clinical trial.
4) To record reasons for non-recruitment and study attrition to inform the design of a larger clinical trial.
5) To measure depression in targeted population to be able to estimate exclusion rates of patients who would be suffering from "Depressive Pseudo Cognitive Impairment" from the future trial.
6) To assess the burden of study-related interventions and assessments on patients and carers.
7) To assess the administration, suitability and adherence of the chosen cognitive method for patients, especially those from ethnic minorities.
8) To assess the administration and suitability of the chosen scales for quality of life measures and activities of daily living in haemodialysis participants.
9) To assess the administration and suitability of the chosen method for measuring carers' burden in this group.

2.2 Outcome

Outcome Measures and Raters Blinding
The primary outcomes related to this feasibility study are overall trial feasibility and mean and variance estimates for Cogstate composite index in the control and possibly intervention arms. Overall feasibility of the trial will be assessed based on recruitment rates and strategy with respect to inclusion and exclusion rates as well as consent rates, adherence to treatment protocol, rate of recruitment after 6 months, withdrawal rates, missing data, and costs of running the study. In addition to this factors that may be related to the outcome (cognitive function) such as age, gender, ethnicity and socioeconomic status will be assessed at baseline. Cognitive function and other outcome measures indicators will be measured at Baseline (0), 6 and 12 months.

3. STUDY DESIGN, METHODS OF DATA COLLECTION AND DATA ANALYIS

The study is a multi-site, prospective, randomised, double-blinded, controlled, feasibility trial with a 1:1 allocation ratio between control and intervention groups. Three local sites, all within the University Hospitals Birmingham NHS Foundation Trust, providing haemodialysis to patients with ESKD will attempt to recruit 90 patients. We will use a mixed method approach, utilising semi-structured interviews, questionnaires and measurement of cognitive function.

The control group will be at the standard dialysate temperature of 36.5 °C (which is the standard of care in the sites), 3 times a week for 12 months. In the intervention group, patients will receive haemodialysis with a dialysate temperature of 35 degree centigrade. The intervention group will start off using a dialysate temperature that is 36 °C. Thereafter the dialysate temperature will be reduced every two week by 0.5 °C until 35 °C or the lowest tolerated temperature reached. Patients who would fail to tolerate the temperature of 35 °C, the lowest tolerated temperature will be carried over to the end of the study.

The main aim of the qualitative component is to assess issues related to patient recruitment. This will include practicalities of implementing cooler dialysate, adherence to treatment, effectiveness of blindness process and identification of factors that may affect routine practice of treatment in various centres. We will apply thematic analysis to qualitative data collected from semi-structured interviews. Interviews will be on a 1:1 basis and will be audio-recorded. They will be transcribed by the research assistant and will be anonymised and securely stored, accessible only by the research team. The purpose of the quantitative component is to estimate the variance of the CogState composite index in the control and treatment groups. This is a feasibility study and statistically or clinically significant changes in outcomes between groups are unlikely, however, a preliminary estimate of a treatment effect is relevant to sample size estimation of future definitive trials.

All outcomes are measured at 0 (baseline), 6 and 12 months by a blinded rater, on a non-dialysis day when the best performance is expected [31]. Blinding of the rater could be compromised if the rater visited the patient during haemodialysis as machine settings might be visible. Testing patients shortly before a haemodialysis treatment might be inconvenient with implications for recruitment and

retention. Therefore, in this study all assessments are conducted in domiciliary visits in the homes of patients or a mutually agreed venue. The carer assessment will also be taken at months 0, 6 and 12 by a blinded independent rater.

All consenting participants eligible for inclusion will be randomised on a 1:1 basis, to the usual or cooler temperature haemodialysis. Randomisation will be stratified by age group and carried out by using Sealed Envelope randomisation software.

This study allows double blinding (i.e., for both patients and investigators). The only person for whom blinding will not be practical is the technical nursing staff setting the temperature of the machine based on the patient's allocation. But they will not have any contact regarding the temperature settings with either patients or investigators performing the assessments regarding the temperature settings. Although patients and raters will not be aware of the temperature setting, some patients may guess that they are at a lower temperature as they will feel colder than usual. However, raters will be strongly instructed not to ask or even encourage a discussion regarding their dialysis temperature during haemodialysis sessions with the patients in the assessments that would take place outside the renal dialysis units. The importance of keeping knowledge and feelings regarding the temperature away from the raters will be fully explained to the patients and advised not to divulge during the psychometric assessment.

4. STUDY SETTING

Patients will be recruited through the renal clinics at Birmingham Heartlands Hospital, Runcorn Road Renal Unit, Solihull Hospital and Castle Vale Renal Unit; all units are under the collective organisation of the University Hospitals Birmingham NHS Foundation Trust.

5. STUDY POPULATION AND RECRUITMENT

5.1 Study population

This study aims to recruit 90 patients; this will be from four participating Haemodialysis units (Birmingham Heartlands Hospital, Solihull Hospital, Runcorn Road and Castle Vale). We plan to recruit 45 patients in the intervention group and 45 patients in the control group with randomisation rate of 1:1 between intervention group and control group.

5.2 Study Interventions

Currently in the UK, temperatures between 36°C and 37°C are empirically used in dialysis; however, the best temperature is not known and may be differentially tolerated depending on the patients' own core temperature [42]. International Clinical guidelines recommend a minimum temperature for the dialysate of 35°C [43,44] mainly for cardiovascular stability. Building on this, the study will aim to investigate the effect of cooled dialysis on cognitive state:

1. An intervention group will use cooled dialysate at temperature of 35°C or the lowest tolerated temperature for 12 months,

2. A control group will use standard dialysate at 36.5°C for 12 months

The study has a double blind randomised control design, the patients and the raters will be blinded. Patients will be randomised to one of two groups: the intervention and the control group. Both groups will have a pre-study run-in phase of two-weeks to establish pre-dialysis temperature with a tympanic thermometer taken at each session. The control group will then use the standard dialysate temperature of 36.5 °C. The intervention group will start off using a dialysate temperature of 36 °C. Thereafter the dialysate temperature will be reduced every two weeks by 0.5 °C until a temperature of 35 °C is reached. Patients who fail to tolerate the temperature of 35 °C, the lowest tolerated temperature will be carried over to the end of the study. Tympanic and dialysate temperature will be recorded at every session regardless of study group to aid data monitoring of the consistency of delivered intervention and allow an interim analysis of patient's temperatures to ensure a clear separation of the study groups. The research nurse will assess temperature tolerability every 2 weeks using "Tolerability of Low Temperature Dialysis Questionnaire" for the first 6 weeks. The patients will not be informed to their group allocation nor the temperature setting of the machine to enable unbiased comparison of the tolerability of the intervention. The investigators carrying out cognitive assessment and study related procedures will also be blinded to their group allocation. The nursing staff must be unblinded in order to deliver the intervention, but any temperature display on the machine will be concealed from the patients. Any patient from the control group and intervention group complaining of feeling cold during haemodialysis session will be provided with an extra blanket to aid tolerance and improve comfort. But if patient could not tolerate the lower temperature to the point that they felt they could terminate the session, the temperature will be increased back to the previous setting.

5.3 Recruitment

Patients will be recruited through the renal clinics at Birmingham Heartlands Hospital, Runcorn Road Dialysis Unit, Solihull Hospital and Castle Vale renal Unit. Patient consent for randomisation and all clinical measurements will be sought in patients who have received at least three (3) months of consecutive dialysis at the point of consent. Three months of haemodialysis treatment has been chosen as this will form our research baseline (month 0 of our research evaluation). This is important because most of the complications and mortality associated with dialysis occur in the first three months of starting treatment.

Suitable patients will be identified by the direct care team. The care team will speak with the suitable patients to see if they are happy to have their details passed on to the research team for further discussion. If the patient agrees, the research team will contact the patient and determine eligibility. Suitable patients will be identified through searching records in the four participating units.

An information pack will be given to the patient (see appendix), who will be given 24 hours to read the information and ask any questions they may have. The research team will then contact the patient, in a pre-agreed manner (i.e. next clinic visit, via telephone) to see if they have any further questions or would like to participate in the study. If they would like to participate in the study they will arrange to meet and complete the consent form (see appendix).

Patients who have just started receiving haemodialysis three-times per week for End-Stage Kidney Disease (ESKD) in one of the four identified dialysis sites; and patients who suffer from End Stage Kidney Disease and expected to need Haemodialysis within six (6) months will be targeted through screening electronic case records after discussion with their treating physician.

Carer Recruitment
Patients, who consent for the study, will be asked to identify the most suitable carer to participate in the study who could be approach in person, over the phone or by post as deemed suitable by the research team and convenient by the carer. Patient's permission to contact their identified carer will be recorded. Carers of consenting patients will be approached in the same manner as above after obtaining patients consent to contact their carers. Patients will still be able to take part in the study even if their carer declines consent.

5.4 Eligibility Criteria

5.4.1 Inclusion criteria

1. Patient is aged ≥18 years.
2. Receiving haemodialysis three (3) times per week for ESKD, for at least 3 months
3. Having proven mental capacity to understand the study and give informed consent

5.4.2 Exclusion criteria

1. Established diagnosis of dementia in a memory clinic or specialised service.
2. Receiving Acetylcholine Esterase Inhibitors
3. Receiving antipsychotic or antidepressants unless stable on treatment for at least 6 weeks
4. Current participation in a study of an investigational medicinal product
5. Inter-current infection
6. An operation date for a living donor kidney transplant within the period of the trial
7. Patients expected to survive less than 1 year according to the treating nephrologist
8. Patients prone to intra-dialytic hypotension or cardiovascular instability during haemodialysis according to the treating nephrologist
9. Patients who are currently taking triptans, dopamine antagonists, tramadol, sedative and opioid analgesics
10. Patients who have a known diagnosis or have other psychiatric conditions, including severe depression, bipolar affective disorder, severe anxiety, panic disorder, substance misuse or psychosis.

11. Currently involved in another intervention study

5.4.3 Inclusion Criteria (Carers)

1. Adult above the age of 18
2. Consents to take part in the study
3. Speaks English

5.4.4 Exclusion Criteria (Carers)

1. Not in regular contact with the patient
2. Any apparent personal or psychological conflicts with the patient that could skew their feedback as judged by the research team.
3. Evidence for very poor physical health that would prevent them from completing the study.

5.5 Participant Withdrawal

Participants are free to withdraw at any time and this will not affect their future care. They will be asked if they wish to withdraw completely, and have all data removed from the study, or just from the point of withdrawal.

5.6 Randomisation

All consenting participants eligible for inclusion will be randomised on a 1:1 basis, to the usual or cooler temperature haemodialysis. Randomisation will be carried out by using Sealed Envelope randomisation software (https://www.sealedenvelope.com/). Randomisation will be stratified by age group (patients under 55 years of age, 55-75 and above 75). Pseudonymisation of data will be applied at the state of data analysis. There will be no identifiable information in the codes produced.

6. DATA COLLECTION

6.1 Baseline Data Collection

Once a participant has consented into the study, baseline data will be collected. This will be collected within 2 weeks of receiving valid informed consent, defined as 0 months.
Data collected at the baseline visit will be demographics (age, gender, ethnicity and socioeconomic status including number of years of education and employment). Confusion Assessment method (CAM) will be used first to rule out Delirium before carrying out the rest of the assessment tools. If CAM is positive, indicating delirium, the rest of the tests for outcome measures will be postponed by two weeks or as directed by the treating clinician. If CAM is negative, the rater will continue to collect the outcome measures as per protocol. Cognitive function (Cogstate, MoCA,, Activities of daily living (Assessment of quality of life, Bristol Activities of daily living, carers Burden assessment, Hospital

anxiety and depression score and heamodialysis recovery time will be collected, as further detailed in section 6.2.

6.2 Follow up Data collection

Data Collection time points are at 6 months and 12 months from baseline assessment with 2 weeks variation window to suit patients social and clinical needs. Throughout the study temperature and Blood pressure records will be recorded as detailed

Outcome Measures and Raters Blinding

The relevant outcomes of this feasibility study are trial feasibility and mean and variance estimates for CogState composite index in the control and possibly intervention arms. Overall feasibility of the trial will be assessed based on recruitment rates and strategy with respect to inclusion and exclusion rates as well as consent rates, adherence to treatment protocol, rate of recruitment after 6 months, withdrawal rates, missing data, and costs of running the study. In addition to these factors that may be related to the outcome (cognitive function) such as age, gender, ethnicity and socioeconomic status will be assessed at baseline. Cognitive function and quality of life indicators will be measured at 0, 6 and 12 months.

All outcomes are measured at 0 (baseline), 6 and 12 months by a blinded, independent rater, on a non-dialysis day, in outpatient setting in MIDRU or other venue chosen by the patient or in a domiciliary visit to patients' own homes if all other options were not suitable for the patient after ensuring staff safety according to University Hospitals Birmingham Foundation Trust policies. In this study all outcome measures assessments are conducted outside the renal dialysis units in order to maintain the rater's blindness, which could be compromised if the rater visited the patient during haemodialysis as machine settings might be visible or patient requiring extra cover. The carer assessment will also be taken at baseline, 6 and 12 months by a blinded rater, which will include scales for carers Burden scale, Quality of life and activities of daily living.
There is a chance that through the study we may identify that patients may have some early cognitive impairment. This information will be communicated to the patient and their GP and any relevant referrals will be made.

Qualitative data and quantitative data to inform future trial design

- We will measure recruitment rates, attrition rates, reasons for non-participation and reasons for study withdrawal.
- We will assess the administration, suitability and adherence of the chosen cognitive and quality of life measures in participants.
- We will assess all patients' experience of being in the study when temperature setting has been stabilised and at the end either by completion or drop out.
- We will use thematic analysis to analyse semi-structured interviews and questionnaires to patients and carers at the end of the study.

Confusion Assessment Method (CAM)

The CAM will be carried out before every cognitive assessment to exclude the effect of delirium on cognitive performance48. The CAM has two parts. Part one is a screen for cognitive impairment. Part two uses four questions to distinguish delirium from persistent cognitive impairment. CAM can be applied to patients from other ethnic minorities as it is based on medical observation.

In this study, this will be used to rule out Delirium that could skew patients' performance on cognitive testing. Therefore, Confusion Assessment method (CAM) will be used first to rule out Delirium before carrying out the rest of the assessment tools. If CAM is positive, indicating delirium, the rest of the tests for outcome measures will be postponed by two weeks or as directed by the treating clinician. If CAM is negative, the rater will continue to collect the outcome measures as below.

Cognitive function

The CogState System

Cognitive assessments such as the Mini-Mental State Examination that offer global cognitive function screening are considerably less sensitive to detecting cognitive functional change and prone to 'practice effects' where participants scores improve due only to test familiarity. The CogState system is a computerized test that assesses a diverse range of key cognitive skills [45]. The CogState system was selected to reduce test fatigue and simplify test administration, whilst preserving strong test-retest reliability (rho=0.81–0.89) [45]. Test scores are computer generated, minimizing human error in administration and scoring, and allowing for exact timing of response speed. It has the advantages of being portable, short (20–30 minutes), game-like in presentation and thus motivating, cross-culturally adaptable and language independent.

The CogState system has been widely validated in large prospective studies of diverse populations including elderly individuals [45]. The CogState system fulfills the criteria recommended for cognitive assessment in clinical trials to employ reliable, sensitive and valid assessment of cognitive functional change[46]. In order to assess the range of human cognitive skills that might be at risk from haemodialysis treatment, we have selected five tests that provide measures of attention, processing speed, visual memory, verbal memory, episodic memory, working memory and executive function. The CogState system provides a rigorous assessment that takes less than 30 minutes to administer. This suits patients with ESKD considering their health state and treatment environment.

CogState is available in 90 languages and uses multiple 'parallel' versions of the tests, thus minimizing practice effects. In regards to patients with ESKD, CogState has many advantages over traditional tools that use lengthy pencil and paper tests such as the Cambridge Cognition Examination (CAMCOG), which make it more suitable to this group of patients.

In this study we will use a battery of tests consists of Detection, Identification, One Back, the International Shopping List Test (immediate and delayed recall) and the Modified Groton Maze Learning Test (Modified GMLT). Raters are trained to use this battery which would take between 20-30 minutes for every patient.

In this study, CogState is used as a detailed measure for cognitive functions.

Montreal Cognitive Assessment (MOCA)

The MoCA is a 30-point test of global cognitive function taking approximately 10 minutes to administer47. There are three alternate forms in English designed to minimize practice effects in longitudinal studies. The MoCA is included primarily to allow comparison with the results of other studies of haemodialysis and ESKD. MOCA is currently validated for the use of haemodialysis patients but in German[55]. We will provide the first English language validation of the MoCA in haemodialysis patients against the CogState.
In this study, this will be used as a global measure for cognitive functions.

Anxiety and depression measurements
The Hospital Anxiety and Depression Scale is a valid measure of anxiety and depression in patients with frequent hospital admissions50. Systematic review identified a cut-off point of 8/21 for anxiety or depression51. Those who score above 15/21 will be referred to appropriate mental health care pathways.
In this study, this will be used to whether patient is suffering from anxiety or depression and its severity.

Quality of life
We will use the Assessment of Quality of Life (AQoL) scale to measure patient's quality of life[49]. AQoL is a generic health-related quality of life instrument, which provides a profile relative to four life dimensions. The administration of AQoL takes 5-10 minutes, by self-administration or by an interviewer. As an observational scale it should be completed by an informant who either speaks English, otherwise, an interpreter will be used to ensure effective communication with Urdu or Bengali speaking families.
In this study, this will be used to measure patient's quality of life.

Activities of daily living
This questionnaire is to be completed by the nominated and consenting carer. The Bristol Activity of Daily Living Scale will be used to measure activities of daily living in relation to cognitive impairment[52]. This is an informant-rated interview of 20 items each rated on 60-point scale. It was designed for use in patients with cognitive impairment. As an observational scale it should be completed by informants who either speak English, or an interpreter will be used for Urdu and Bengali speaking families.
In this study, this will be used to measure patient's levels of activities of daily living.

Carer Burden Assessment
This questionnaire is to be completed by the nominated and consenting carer. We will measure carer burden using Caregiver Burden scale, which was developed to assess perceived burden among caregivers of family members with cognitive impairment[53]. Communication with families from ethnic minorities will be in English if deemed possible or relevant interpreters will be used.
In this study, this will be used to carers care burden.

Tolerability of Low Temperature

This is assessed by "Tolerability of Low Temperature Dialysis Questionnaire" which is a simple questionnaire that takes about 3 minutes to complete. Patients are asked about their ability to tolerate the low temperature and their level of comfort. They are also asked whether they need any extra support. The questionnaire was designed at Birmingham Heartlands Hospital and used for a pilot study where it showed face validity and reliability. It appeared to be user friendly.

Intradialytic hypotension measurements

Intradialytic hypotension is an important explanatory outcome for this trial as this is thought to be the main mechanism by which dialysate cooling might prevent cognitive decline. Intradialytic hypotension has been variably defined in prior studies making comparisons difficult. Symptoms are frequently not reported by patients who are hypotensive during hemodialysis, which leads to an underestimation of Intradialytic hypotension if symptom-based definitions are used[53]. In a recent 77 patient study, thresholds that maximized the probability of a nursing intervention rather than a session remaining asymptomatic were systolic Blood Pressure (BP) <100 mmHg or a 20% reduction in systolic BP from baseline[53]. The largest study examined the association of various definitions of intradialytic hypotension on mortality in >10,000 haemodialysis patients from 2 clinical trials[54]. This showed that intradialytic hypotension definitions based on patient symptoms, nursing interventions or decreases in BP during dialysis were not associated with increased mortality whilst absolute systolic BP <90mm Hg was potently associated with greater mortality[54]. Of relevance to cognitive function, recent data demonstrates that that brain ischemia can occur at a variety of thresholds that would not typically be recognized as intradialytic hypotension[55]. Therefore, in the absence of cerebral perfusion monitoring a pragmatic solution is to record BP and apply both a nadir-based definition of intradialytic hypotension as well as relative change using routinely recorded BP data. For this study, BP will be recorded before and after dialysis in the same way as routine clinical practice by the clinical team. For analysis, intradialytic hypotension will be defined as systolic BP during dialysis >20% from baseline or <90mmHg. Nursing interventions for intradialytic hypotension (slowing down ultrafiltration, giving additional fluid) will be recorded.

Blood Pressure and Intradialytic Hypotension

- Blood pressure; measured at baseline before and after each dialysis session,
- Symptomatic Intradialytic hypotension as recorded as 'Crash' in electronic renal dialysis records
- Nursing interventions for intradialytic hypotension e.g. need for saline infusion, stoppage of dialysis

Other physiological measurements (measured three-times weekly during haemodialysis as part of routine care)

- Body temperature before and after each session
- Pulse rate before and after each session
- Interdialytic weight gain 1 month preceding the date of assessment

Laboratory measurements (measured monthly as part of routine care, collected from patients electronic medical records)

- KT/V as markers of adequate solute clearance
- Routine haematology and biochemistry, FBC, U&E, Creatinine, Calcium and phosphate
- Routine blood samples that are analysed will not be stored for research purposes and will be destroyed in accordance with University Hospitals Birmingham's Laboratory standard operating procedures.

Haemodialysis recovery time

This is assessed by a simple question, "How long does it take you to recover from a dialysis session''. In a study of 6860 patients in 12 countries, longer self-reported recovery time is independently associated with reduced health-related quality-of-life, increased hospitalisation and reduced survival 54. It will be assessed at baseline, 6 months and 12 months.

Table 1. Schedule of data collection

	Baseline (O month)	6 months	12 months
Consent	X		
Randomisation	X		
Baseline data (defined)	X		
Lower Temperature tolerance	X[$]		
Cognitive function:<br>Cogstate<br>MoCA<br>CAM	 X<br>X<br>X	 X<br>X<br>X	 X<br>X<br>X
Tolerability of Low Temperature Dialysis Questionnaire	Every two weeks at the first 6 weeks only. (the lowest tolerated temp will be carried over to the end of the study)		
Residual renal function	X	X	X
Activities of daily living:<br>Assessment of QoL<br>Bristol ADL	 X<br>X	 X<br>X	 X<br>X
Carer burden assessment	X	X	X
HADS	X	X	X
Haemodialysis recovery time	X	X	X
Qualitative interview			At completion or drop put
Dialysis temperature recording	During each dialysis session		

Physiological measurements*	During each dialysis session		
Laboratory measurements**	Measured monthly as part of routine care		
Report AE/SAEs	X	X	X
Review Concomitant Medications	X	X	X

* Blood pressure (pre and post haemodialysis), intradialytic hypotension, nursing interventions for intradialytic hypotension, intradialytic weight gain over preceding 1 month,
** KT/V as markers of adequate solute clearance, routine haematology and biochemistry
[\$] Will be monitored every two weeks for the first six weeks

6.3 Data Management and Analysis

6.3.1 Sample Size

The outcome data from this feasibility study will be used to inform the sample size calculation for the definitive trial, by providing estimates of the outcome (and its variability) in the control and intervention arms, and give an indication of the expected attrition. The study aims to recruit a total of 90 patients from four sites. Lancaster et al, 2004[56], outlined the key aspects of feasibility studies, and indicated at least 30 patients per each arm are required to identify the sample variability (standard deviation) in key variables to enable the calculation of power for testing hypotheses in subsequent definitive studies.
The primary outcome in the definitive study is likely to be a value from the Montreal Cognitive Assessment (MoCA). With 45 patients in each arm, and assuming that the mean (SD) value of MoCA is 27 (2) in the control and intervention arms at the study start, we could expect a 95% confidence interval to range from 26.4 to 27.6 in each arm. This will give adequate precision for the estimate required in the study. With 45 patients in the control arm, and assuming a mean (SD) value of MoCA of 22 (3) after 12 months, we could expect a 95% confidence interval to range from 21.1 to 22.9. In the intervention arm, assuming a mean (SD) MoCA value of 25 (3) after 12 months, we could expect a 95% confidence interval to range from 24.1 to 25.9. Furthermore, with a total sample size of 90 patients, with an expected loss of 20% of the patients, a 95% confidence interval could be produced, ranging from 70.2% to 87.7%.

6.3.2 Data management

Data will be entered and stored in a password protected electronic excel spread sheet, their research study number will be included and any patient identifiable information removed. The original paper copies of all study related data will be treated as confidential documents and held securely in accordance with regulations. Participants will each be assigned a unique trial identity code for use on all study documents. All source documents will be filed at the principal investigator's site. All databases will be held on University Hospitals Birmingham NHS Foundation Trust secure servers, in accordance with the Trust data protection policies.

6.3.3 Data analysis

The analysis of the data centres around the summary of the outcome, to inform the sample size calculation for the definitive study. The baseline characteristics, the baseline outcome values, and the follow-up outcome values will be summarised using appropriate summary statistics. For continuous outcomes, this will be either mean and standard deviation or median and interquartile range. Binary and categorical outcomes will be presented as numbers and percentages. The primary aim of the analysis is to estimate the mean and standard deviation for MoCA at baseline and follow-up in both trial arms, and obtain an estimate of the attrition. For all analysis, the level of significance will be set at 5%, so that 95% confidence intervals will be presented.

As an exploratory analysis, we will conduct a complete case analysis of the primary and secondary outcomes. A linear regression model will be used for continuous outcomes (e.g. MoCA) and a logistic regression model will be used for binary outcomes. Each model will include the baseline measurement and treatment arm as independent variables. In case of observing very positive results, an interim analysis would be arranged after 50% of the patients completed the study to inform the future direction of this feasibility study and further application for a defensive study if there were enough statistical power to achieve the set objectives. All analysis will be conducted in Stata 15.

7. SAFETY & ADVERSE EVENT MANAGEMENT

7.1 Definitions

7.1.1 Adverse Events (AE)

An adverse event is defined as any untoward medical occurrence in a subject and which does not necessarily have a causal relationship with this treatment.
Adverse reactions (AR)
An adverse reaction is defined as any untoward and unintended response to the study intervention. A causal relationship between the trial treatment and an adverse event is at least a reasonable possibility, ie the relationship cannot be ruled out.

7.1.2 Serious Adverse Events (SAEs)

A serious adverse event is an AE that fulfils one or more of the following criteria:

- Results in death
- Is immediately life-threatening
- Requires hospitalisation or prolongation of existing hospitalisation
- Results in persistent or significant disability or incapacity
- Is otherwise medically significant (e.g. important medical events that may not be immediately life-threatening or result in death or hospitalisation but may jeopardise the patient or may require intervention to prevent one of the other outcomes listed above
- Involves a Fistula Thrombosis, Myocardial Infarction, Stroke and/or Angina.

7.1.3 Serious Adverse Reactions (SARs)

A SAR is defined as an SAE that has a definite, probable or possible causal relationship to the study intervention. The causality of SAEs (i.e., relationship to intervention will be assessed by the investigator(s) on the SAE form.

Suspected Unexpected Serious Adverse Reactions (SUSARs)
Suspected Unexpected Serious Adverse Reactions (SUSARs) are SARs that are also unexpected i.e. their nature or severity is not with haemodialysis and are considered to be caused by the intervention.

7.2 Reporting Procedures

7.2.1 Causality

The PI or other delegated site investigators must perform an evaluation of causality for each adverse event.
Causal relationship to the trial treatment must be determined as follows:
• **None** - There is no evidence of any causal relationship.

• **Unlikely** - There is little evidence to suggest a causal relationship (e.g. because the event did not occur within a reasonable time after administration of the trial treatment). There is another reasonable explanation of the event (e.g. the patient's clinical condition, other concomitant medications).
• **Possible** - There is some evidence to suggest a causal relationship (e.g. because the event occurs within a reasonable time after administration of the trial treatment). However, the influence of other factors may have contributed to the event (e.g. the patient's clinical condition, other concomitant medications).
• **Probable** - There is evidence to suggest a causal relationship and the influence of other factors is unlikely.
• **Definitely** - There is clear evidence to suggest a causal relationship and other possible contributing factors can be ruled out.

7.2.2 Reporting ARs

All Adverse Reactions that occur between the first administration of study intervention and 30 days post last study intervention must be recorded in the trial CRFs, together with data including date of onset and resolution, outcome, severity and causality for the trial intervention.
.

7.2.3 Reporting SAEs, SAR's and SUSARs

SAEs and SUSARs will be reported using the SAE form in the patient's CRF. The Principal Investigator in each centre must report any SAEs and SUSARs to the Trial Co-ordinating Centre within 24 hours of them becoming aware of it.
The SAE form should be completed and faxed to the R&D department at 0121 424 3167. The trial co-ordinator will liaise with the Investigator to compile all the necessary information. The Trial Co-ordinating Centre is responsible for reporting adverse events to the sponsor, ethics committee within required timelines.

8. AUDIT & MONITORING

The study may be monitored and/or audited by University Hospitals Birmingham NHS Foundation Trust under their remit as Sponsor and other regulatory bodies to ensure adherence to Good Clinical Practice and the UK Policy Framework for Health and Social Care Research.

Monitoring of study data shall include confirmation of subject eligibility and informed consent; adherence to the study protocol, source data verification; data storage and data transfer procedures; local quality control checks and procedures, back-up and disaster recovery of any local databases and validation of data manipulation. Study conduct will be subject to systems audit of the trial master file for inclusion of essential documents; permissions to conduct the trial; study delegation log; CV.s of study staff and training received; local document control procedures; consent procedures and recruitment logs; adherence to procedures defined in the protocol (e.g. inclusion / exclusion criteria, timeliness of visits); accountability of study materials and equipment calibration logs. The Sponsors QA Manager, or a nominated designee of the Sponsor, shall carry out monitoring of study data as an on-going activity.

Study data and evidence of monitoring and systems audits will be made available for inspection by the regulatory authority as required.

9. STUDY MANAGEMENT

The conduct of the research will be overseen by the Chief Investigator and a representative of the Sponsor from University Hospitals Birmingham NHS Foundation Trust. The Sponsor representative will report into the R&D Management Team on progress of the trial, in line with the Trust's standard operating procedures for Sponsorship.
The trial management committee (TMC) will meet at least quarterly during the duration of the study. They will provide guidance on the day to day running of the study, review study aims and ensure they are being met, they will report into the Trial Steering Committee (TSC).

The trial steering committee, will be independent from the TMC, with the exception of a Sponsor representative. The TSC will meet at least every 6 months to review study data and offer guidance on the study outcomes and further direction of the potential full study.

10. REGULATORY APPROVALS

Approvals will be obtained from the Research Ethics Committee (REC) and Health Research Authority (HRA) prior to the commencement of this study. Confirmation of Capability and Capacity will be obtained from the relevant Trust prior to any research activity being undertaken.
The study will be conducted in accordance with principles of the International Committee on Harmonisation and Good Clinical Practice Guidelines.

10.1 Sponsorship and Indemnity

University Hospitals Birmingham NHS Foundation Trust will act as the Sponsor to this study. Delegated responsibilities will be assigned to the Chief Investigator.
University Hospitals Birmingham NHS Foundation Trust holds standard NHS Hospital indemnity and insurance cover with NHS Litigation Authority for NHS Trusts in England, which apply to this study.

11. PATIENT AND PUBLIC INVOLVEMENT

We had strong involvement from service users in designing the project as well as the management of this project. Mr Samir Youseff who is a co-applicant and co-designer of this project is suffering from ESKD and has been receiving haemodialysis (HD) with cooled dialysis fluid for many years. He is very supportive to our project and keen to contribute to improvement of HD practice for the benefits of all patients who suffer from the same disease. He has first hand experience of the concepts of our research and possible barriers. Mr Samir Youssef has shaped and refined the design of the study in many ways. He used his experience to develop and refine our project. He helped write the plain

English summary and has been involved in every step of the discussion, especially concerning the appropriateness of the study design from a patient perspective. The idea of scheduling cognitive assessments to be carried out at patients' homes or other mutually agreed venue on the day following HD session was primarily due to his involvement and guidance. He advised us that patients would not like to take the assessment during the session as they feel tired and prefer to rest or listen to music, and would not come back to hospital for an additional visit.
We have strong links with the University Hospitals Birmingham NHS Foundation Trust Clinical Research Ambassadors Group (CRAG) to ensure that our study will remain patient centered and workable for all the participants. Our service users representative (Mr Samir Youssef) will lead the PPI advisory group for the feasibility and the potential future definitive study. Mr Samir Youssef will take an active role in developing patient information and will act as a contact point for patients throughout the study.

12. PROTOCOL COMPLIANCE

Any deviations from dialysis temperature will be recorded in the CRF, with a brief note of what temperature and reason why this used. This will be reviewed and assessed, from a clinical perspective by a renal physician to ensure patient safety. Should there be any deviation from the protocol in any way, the sponsor shall be notified of what has been done, and the sponsor will ensure actions are in place so that this does recur, and that all members part of the study are fully compliant to the protocol.

13. AMENDMENTS

If any amendments to the study are required, the amendment will be agreed by the trial management committee and approved by the Sponsor. The sponsor will classify the amendments as either substantial or non-substantial in accordance with the relevant guidance. The appropriate approvals from the relevant regulatory authorities will be obtained and once received the amendment will be implemented. A full audit trail of the amendment will be contained in the Trial Master File.

14. DISSEMINATION POLICY

The findings of this feasibility will be reported at appropriate conferences and the aim is to publish them in an relevant open access publication. The dissemination of knowledge generated and findings will be reported and a summary report will be produced adherent to the funder's guidelines at the completion of the project.

14.1 Authorship eligibility guidelines and any intended use of professional writers

All Co-applicants and members of the trial management committee will be named as authors on the paper. The lead author will be the chief investigator of the study. The subsequent order of authorship will be determined based upon the level of contribution in writing the publication.

15 REFERENCES

1. Chiu Y-W, Teitelbaum I, Misra M, de Leon EM, Adzize T, Mehrotra R. Pill Burden, Adherence, Hyperphosphatemia, and Quality of Life in Maintenance Dialysis Patients. Clinical Journal of the American Society of Nephrology 2009;4:1089-96.
2. Steenkamp R, Shaw C, Feest T. UK Renal Registry 15th annual report: Chapter 5 survival and causes of death of UK adult patients on renal replacement therapy in 2011: national and centre-specific analyses. Nephron Clinical practice 2013;123 Suppl 1:93-123.
3. Bradbury BD, Fissell RB, Albert JM, et al. Predictors of Early Mortality among Incident US Hemodialysis Patients in the Dialysis Outcomes and Practice Patterns Study (DOPPS). Clinical Journal of the American Society of Nephrology 2007;2:89-99.
4. Mapes DL, Lopes AA, Satayathum S, et al. Health-related quality of life as a predictor of mortality and hospitalization: the Dialysis Outcomes and Practice Patterns Study (DOPPS). Kidney international 2003;64:339-49.
5. Hedayati SS, Bosworth HB, Briley LP, et al. Death or hospitalization of patients on chronic hemodialysis is associated with a physician-based diagnosis of depression. Kidney Int 2008;74:930-6.
6. Griva K, Stygall J, Hankins M, Davenport A, Harrison M, Newman SP. Cognitive Impairment and 7-Year Mortality in Dialysis Patients. American Journal of Kidney Diseases 2010;56:693-703.
7. Moens K, Higginson IJ, Harding R, Euro I. Are There Differences in the Prevalence of Palliative Care-Related Problems in People Living With Advanced Cancer and Eight Non-Cancer Conditions? A Systematic Review. Journal of Pain and Symptom Management 2014;48:660-77.
8. Ramkumar N, Beddhu S, Eggers P, Pappas LM, Cheung AK. Patient preferences for in-center intense hemodialysis. Hemodialysis international International Symposium on Home Hemodialysis 2005;9:281-95.
9. Kimmel PL, Cohen SD, Weisbord SD. Quality of life in patients with end-stage renal disease treated with hemodialysis: survival is not enough! J Nephrol 2008;21 Suppl 13:S54-8.
10. Wanner C, Krane V, März W, et al. Atorvastatin in Patients with Type 2 Diabetes Mellitus Undergoing Hemodialysis. New England Journal of Medicine 2005;353:238-48.
11. Pfeffer MA, Burdmann EA, Chen C-Y, et al. A Trial of Darbepoetin Alfa in Type 2 Diabetes and Chronic Kidney Disease. New England Journal of Medicine 2009;361:2019-32.
12. Investigators TET. Effect of Cinacalcet on Cardiovascular Disease in Patients Undergoing Dialysis. New England Journal of Medicine 2012;367:2482-94.
13. Kurella M, Chertow GM, Fried LF, et al. Chronic kidney disease and cognitive impairment in the elderly: the health, aging, and body composition study. J Am Soc Nephrol 2005;16:2127-33.
14. Davey A, Elias MF, Robbins MA, Seliger SL, Dore GA. Decline in renal functioning is associated with longitudinal decline in global cognitive functioning, abstract reasoning and verbal memory.

Nephrology, dialysis, transplantation : official publication of the European Dialysis and Transplant Association - European Renal Association 2013;28:1810-9.
15. Sedaghat S, Vernooij MW, Loehrer E, et al. Kidney Function and Cerebral Blood Flow: The Rotterdam Study. Journal of the American Society of Nephrology 2015.
16. Murray AM, Tupper DE, Knopman DS, et al. Cognitive impairment in hemodialysis patients is common. Neurology 2006;67:216-23.
17. Schneider SM, Kielstein JT, Braverman J, Novak M. Cognitive Function in Patients With Chronic Kidney Disease: Challenges in Neuropsychological Assessments. Seminars in nephrology 2015;35:304-10.
18. Drew DA, Weiner DE. Cognitive impairment in chronic kidney disease: keep vascular disease in mind. Kidney international 2014;85:505-7.
19. Lu R, Kiernan MC, Murray A, Rosner MH, Ronco C. Kidney-brain crosstalk in the acute and chronic setting. Nature Reviews Nephrology 2015;11:707-19.
20. Miwa K, Tanaka M, Okazaki S, et al. Chronic kidney disease is associated with dementia independent of cerebral small-vessel disease. Neurology 2014;82:1051-7.
21. Lisowska-Myjak B. Uremic toxins and their effects on multiple organ systems. Nephron Clinical practice 2014;128:303-11.
22. Kielstein H, Suntharalingam M, Perthel R, et al. Role of the endogenous nitric oxide inhibitor asymmetric dimethylarginine (ADMA) and brain-derived neurotrophic factor (BDNF) in depression and behavioural changes: clinical and preclinical data in chronic kidney disease. Nephrol Dial Transplant 2015;30:1699-705.
23. Odudu A, McIntyre C. Influence of dialysis therapies in the development of cardiac disease in CKD. J Ren Care 2010;36 Suppl 1:47-53.
24. Dubin RF, Teerlink JR, Schiller NB, Alokozai D, Peralta CA, Johansen KL. Association of segmental wall motion abnormalities occurring during hemodialysis with post-dialysis fatigue. Nephrol Dial Transplant 2013;28:2580-5.
25. Prohovnik I, Post J, Uribarri J, Lee H, Sandu O, Langhoff E. Cerebrovascular effects of hemodialysis in chronic kidney disease. Journal of Cerebral Blood Flow and Metabolism 2007;27:1861-9.
26. Flythe JE, Xue H, Lynch KE, Curhan GC, Brunelli SM. Association of Mortality Risk with Various Definitions of Intradialytic Hypotension. Journal of the American Society of Nephrology 2014.
27. Murray AM, Pederson SL, Tupper DE, et al. Acute variation in cognitive function in hemodialysis patients: a cohort study with repeated measures. Am J Kidney Dis 2007;50:270-8.
28. Wolfgram DF, Szabo A, Murray AM, Whittle J. Risk of dementia in peritoneal dialysis patients compared with hemodialysis patients. Perit Dial Int 2015;35:189-98.
29. Selby NM, McIntyre CW. Peritoneal dialysis is not associated with myocardial stunning. Perit Dial Int 2011;31:27-33.
30. Stefansson BV, Brunelli SM, Cabrera C, et al. Intradialytic hypotension and risk of cardiovascular disease. Clin J Am Soc Nephrol 2014;9:2124-32.
31. Chertow GM, Levin NW, Beck GJ, et al. In-center hemodialysis six times per week versus three times per week. The New England journal of medicine 2010;363:2287-300.
32. Jassal SV, Devins GM, Chan CT, Bozanovic R, Rourke S. Improvements in cognition in patients converting from thrice weekly hemodialysis to nocturnal hemodialysis: a longitudinal pilot study. Kidney Int 2006;70:956-62.
33. Maggiore Q, Pizzarelli F, Zoccali C, Sisca S, Nicolo F, Parlongo S. Effect of extracorporeal blood cooling on dialytic arterial hypotension. Proceedings of the European Dialysis and Transplant Association European Dialysis and Transplant Association 1981;18:597-602.

34. Toth-Manikowski SM, Sozio SM. Cooling dialysate during in-center hemodialysis: Beneficial and deleterious effects. World Journal of Nephrology 2016;5:166-71.
35. Roumelioti ME, Unruh ML. Lower Dialysate Temperature in Hemodialysis: Is It a Cool Idea? Clin J Am Soc Nephrol 2015.
36. van der Sande FM, Wystrychowski G, Kooman JP, et al. Control of Core Temperature and Blood Pressure Stability during Hemodialysis. Clinical Journal of the American Society of Nephrology : CJASN 2009;4:93-8.
37. Mustafa RA, Bdair F, Akl EA, et al. Effect of Lowering the Dialysate Temperature in Chronic Hemodialysis: A Systematic Review and Meta-Analysis. Clinical Journal of the American Society of Nephrology 2015.
38. Eldehni MT, Odudu A, McIntyre CW. Randomized clinical trial of dialysate cooling and effects on brain white matter. Journal of the American Society of Nephrology : JASN 2015;26:957-65.
39. Eldehni MT, Odudu A, McIntyre CW. Characterising Haemodynamic Stress during Haemodialysis Using the Extrema Points Analysis Model. Nephron Clinical Practice 2014;128:39-44.
40. Odudu A, Eldehni MT, McCann GP, McIntyre CW. Randomized Controlled Trial of Individualized Dialysate Cooling for Cardiac Protection in Hemodialysis Patients. Clinical Journal of the American Society of Nephrology 2015.
41. Bowen DJ KM, Spring B, Cofta-Woerpel L, Linnan L, Weiner D, Bakken S, Kaplan CP, Squiers L, Fabrizio C, Fernandez M. . How we design feasibility studies. American journal of preventive medicine 2009;36:452-7.
42. Passlick-Deetjen J, Bedenbender-Stoll E. Why thermosensing? A primer on thermoregulation. Nephrol Dial Transplant 2005;20:1784-9.
43. Tattersall J, Martin-Malo A, Pedrini L, et al. EBPG guideline on dialysis strategies. Nephrology Dialysis Transplantation 2007;22:ii5-ii21.
44. K/DOQI clinical practice guidelines for cardiovascular disease in dialysis patients. Am J Kidney Dis 2005;45:S1-153.
45. Maruff P, Thomas E, Cysique L, et al. Validity of the CogState brief battery: relationship to standardized tests and sensitivity to cognitive impairment in mild traumatic brain injury, schizophrenia, and AIDS dementia complex. Archives of clinical neuropsychology : the official journal of the National Academy of Neuropsychologists 2009;24:165-78.
46. Mohs RC, Knopman D, Petersen RC, et al. Development of Cognitive Instruments for Use in Clinical Trials of Antidementia Drugs: Additions to the Alzheimer's Disease Assessment Scale That Broaden Its Scope. . Alzheimer Disease & Associated Disorders 1997;11.
47. Nasreddine ZS, Phillips NA, Bédirian V, et al. The Montreal Cognitive Assessment, MoCA: a brief screening tool for mild cognitive impairment. Journal of the American Geriatrics Society 2005;53:695-9.
48. Nouye SK VC, Alessi CA. Clarifying confusion: The Confusion Assessment Method. A new method for detecting delirium. . Annual Intern Med 1990;113:941-8.
49. Hawthorne G RJ, & Osborne R. The Assessment of Quality of Life (AQoL) instrument: a psychometric measure of health-related quality of life. . Quality of Life Research 1999;8:209-24.
50. Zigmond AS, Snaith RP. The hospital anxiety and depression scale. Acta psychiatrica scandinavica 1983;67:361-70.
51. Bjelland I DA, Haug TT, & Neckelmann D. The validity of the Hospital Anxiety and Depression Scale: an updated literature review. Journal of psychosomatic research 2002;52:69-77.
52. Bucks RS AD, Wilcock GK, & Siegfried K. Assessment of activities of daily living in dementia: development of the Bristol Activities of Daily Living Scale. Age and ageing 1996;25:113-20.

53. Macera CA EE, Jannarone RJ, Davis DR, Stoskopf CH. A Measure of Perceived Burden among Caregivers. Evaluation & the Health Professions 1993;16:204-11.
54. Rayner HC, Zepel L, Fuller DS, et al. Recovery time, quality of life, and mortality in hemodialysis patients: the Dialysis Outcomes and Practice Patterns Study (DOPPS). Am J Kidney Dis 2014;64:86-94.
